# Molecular mechanisms and therapeutic strategies for neuromuscular diseases

**DOI:** 10.1007/s00018-024-05229-9

**Published:** 2024-04-28

**Authors:** Alberto Andrea Zambon, Yuri Matteo Falzone, Alessandra Bolino, Stefano Carlo Previtali

**Affiliations:** 1 Division of Neuroscience, IRCCS San Raffaele Scientific Institute, Institute for Experimental Neurology, Inspe, Milan, Italy; 2 Neurology Department, San Raffaele Scientific Institute, Milan, Italy; 3 Vita-Salute San Raffaele University, Milan, Italy

**Keywords:** Myopathy, Neuropathy, Neuromuscular junction, Motor neuron disease, Therapy

## Abstract

Neuromuscular diseases encompass a heterogeneous array of disorders characterized by varying onset ages, clinical presentations, severity, and progression. While these conditions can stem from acquired or inherited causes, this review specifically focuses on disorders arising from genetic abnormalities, excluding metabolic conditions. The pathogenic defect may primarily affect the anterior horn cells, the axonal or myelin component of peripheral nerves, the neuromuscular junction, or skeletal and/or cardiac muscles. While inherited neuromuscular disorders have been historically deemed not treatable, the advent of gene-based and molecular therapies is reshaping the treatment landscape for this group of condition. With the caveat that many products still fail to translate the positive results obtained in pre-clinical models to humans, both the technological development (e.g., implementation of tissue-specific vectors) as well as advances on the knowledge of pathogenetic mechanisms form a collective foundation for potentially curative approaches to these debilitating conditions. This review delineates the current panorama of therapies targeting the most prevalent forms of inherited neuromuscular diseases, emphasizing approved treatments and those already undergoing human testing, offering insights into the state-of-the-art interventions.

## Introduction

Neuromuscular diseases comprise a spectrum of disorders affecting motor neurons in the spinal cord, sensory neurons in the dorsal root ganglia, peripheral nerves, neuromuscular junction and/or skeletal muscles. Cranial nerves (and their nuclei) as well as components of the vegetative system can be also affected. These diseases mainly compromise motricity and sensation and may be a consequence of many different causes including acquired and genetic factors. While for most of the acquired neuromuscular diseases a progressive number of therapies have been discovered and are now used in clinical practice, there is still a significant unmet need for the development of curative treatments for inherited and degenerative forms. This landscape is rapidly changing though, with therapies already available in clinical practice and others under evaluation in clinical trials or preclinical studies.

**Fig. 1:**
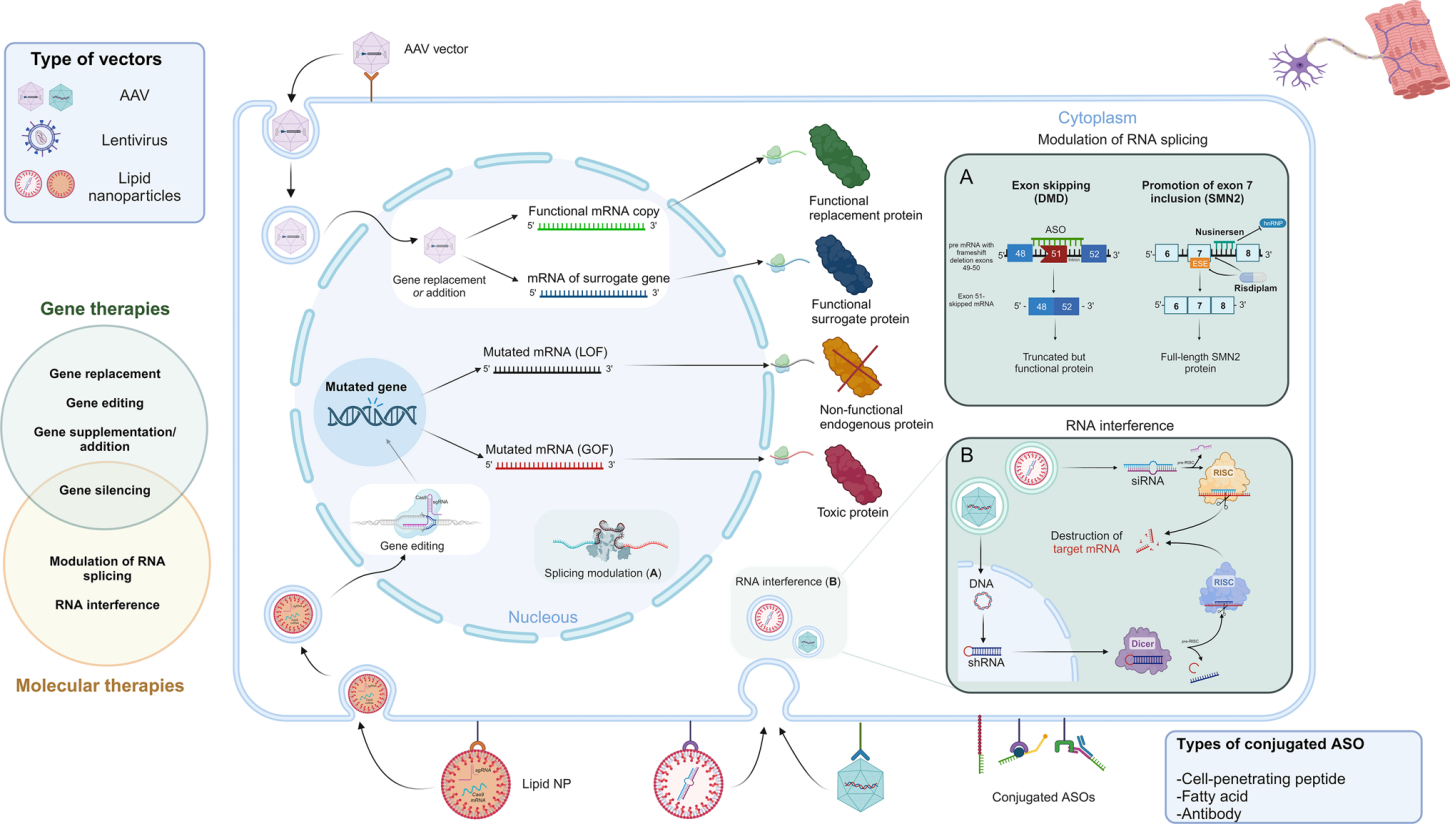
Schematic representation of the therapeutic landscape applicable to neuromuscular diseases, with a focus on gene and molecular therapies. Treatment strategies vary according to the underlying genetic defect, namely loss of function (LOF) or gain of function (GOF) mutations (depicted in the centre of the figure). A defective gene may be corrected at DNA level via gene editing/silencing obtained, for instance, using Crispr/Cas9 technology, with still important concerns regarding possible off-target events. Alternatively, coding (c)DNA containing a tissue-specific promoter and a gene cassette encoding for a functional or surrogate protein can be delivered by viral vectors and be subsequently translated by the cell apparatus. The persistence of cDNA in time may vary according to cell type. Molecular therapies can act at RNA level either by modulating splicing or by altering messenger (m)RNA transcription. The former strategy is usually obtained using antisense oligonucleotides (ASO) (**A**), as the ones developed for exon skipping in Duchenne muscular dystrophy or to counteract the splicing of exon 7 of the *SMN2* gene in spinal muscular atrophy. Of note, ASO can be conjugated with cell-penetrating peptides, fatty acids, or specific antibodies to increase their delivery to target organs, in order to augment efficacy while reducing potential side effects. The modulation of RNA transcription can be obtained by single-stranded ASO or by double stranded short interfering (si)RNA. The latter can be either directly delivered to cells or processed from short/small hairpin (sh)RNA (**B**). Lastly, both gene and molecular therapies can be delivered by different viral and non-viral vectors (including nanoparticles and cells), which are being constantly improved

The most compelling therapeutic approaches for inherited disorders can be delineated as follows (see also Fig. 1):

- Treatments designed to rectify genetic anomalies at the DNA level, including gene replacement, gene editing, and gene addition (i.e., the provision/upregulation of proteins that compensate for the mutant gene); these can be termed “gene therapies”.
- Molecular therapies directed at modulating RNA transcripts, such as the use of antisense oligonucleotides (ASO) or small molecules to promote alternative splicing or the implementation of double-stranded, small interfering RNA (siRNA) to suppress pathogenic variants that cause toxic gain of function.
- Infusion of progenitor cells (cell therapies) to replace or modulate the function of damaged cells, a strategy that is currently more suitable for skeletal muscle.
- Interventions aimed at post-translational modifications of specific proteins, exemplified by post-translational glycosylation of α-dystroglycanopathies.
- Treatments targeting one or multiple downstream effects including inflammation, fibrosis, excessive reactive oxygen species, membrane instability, protein aggregates accumulation etc.

Both gene and molecular therapies can be delivered by viral or non-viral vectors (such as lipid nanoparticles or engineered cell lines), each characterized by a specific profile of loading capacity, tissue trophism/specificity etc. Cellular therapy can be utilized both for its direct effects and as a carrier of therapeutic products.

In this review, we will describe the state of the art of the therapies for the most common forms of neuromuscular diseases that have been reported in the literature, excluding acquired and metabolic conditions. Main ongoing clinical trials are reported in Table 1.

**Table 1:** Therapies for inherited neuromuscular disorders that have reached human application

	Primary Target	Disease	Compound	Mechanism of action	Delivery and dosing	Clinical trial/status	Clinical trials registry identifier
Gene therapies							
Gene replacement	Brain and motor neuron	GAN	scAAV9/JeT-GAN	AAV9-delivered *GAN*	*IT, single dose*	Phase I	NCT02362438
	Motor neuron	SMA 1	Onasemnogene abeparvovec	AAV9-delivered *SMN1*	*i.v., single dose*	EMA/FDA approval	
	Motor neuron	SMARD/CMT	IGHMBP2	AAV9-delivered *IGHMBP2*	*IT, single dose*	Phase I/II	NCT05152823
	Muscle	X-MTM	AT132	AAV8-delivered *MTM1*	*i.v., single dose*	Phase I/II/III	NCT03199469
	Muscle	LGMD2B/R2	SRP-6004	AAVrh74-delivered *DYSF*	*i.v., single dose*	Phase I	NCT05906251
	Muscle	LGMD2D/R3	SRP-9004	AAVrh74-delivered α*-SG*	*i.v., single dose*	Phase I/IIA	NCT01976091
	Muscle	LGMD2E/R4	SRP-9003	AAVrh74-delivered β*-SG*	*i.v., single dose*	Phase I/II/III	NCT05876780
	Muscle	LGMD2C/R5	ATA-200	AAV8-delivered γ-*SG*	*i.v., single dose*	Phase I/II	NCT05973630
	Muscle	LGMD2I/​R9	GNT0006	AAV8-delivered *FKRP*	*i.v., single dose*	Phase I/II	NCT05224505
	Muscle	LGMD2I/​R9	LION-101	AAV-delivered *FKRP*	*i.v., single dose*	Phase I/II	NCT05230459
Gene editing	Liver	ATTRv	NTLA-2001	CRISPR/Cas9 editing	*i.v., single dose*	Phase I	NCT04601051
Gene addition	Muscle	DMD	Delandistrogene moxeparvovec	AAVrh74-delivered micro-dys	*i.v., single dose*	FDA conditional approval; Phase III	NCT05096221
	Muscle	DMD	PF-06939926	AAV9-delivered mini-dys	*i.v., single dose*	Phase III	NCT03362502
	Muscle	DMD	SGT-003	AAV9-delivered micro-dys	*i.v., single dose*	Phase I/II	NCT06138639
	Muscle	DMD	RGX-202	AAV8-delivered micro-dys	*i.v., single dose*	Phase I/II	NCT05693142
	Muscle	DMD	RAAVRH74.MCK.GALGT2	rAAVrh74-delivered *GALGT2* gene	*i.v., single dose*	Phase I/II	NCT03333590
Molecular therapies							
RNA splicing modulation	Motor neuron	SMA 1,2,3	Nusinersen	ASO (*SMN2*)	IT, e*very 4 m*	EMA/FDA approval	
	Motor neuron	SMA 1,2,3	Risdiplam	Small molecule (*SMN2)*	*Oral, daily*	EMA/FDA approval	
	Muscle	DMD	Eteplirsen	ASO, exon 51 skipping	*i.v., weekly*	FDA conditional approval	
	Muscle	DMD	SRP-5051	ASO, exon 51 skipping	*i.v., monthly*	Phase III	NCT04004065
	Muscle	DMD	Casimersen	ASO, exon 45 skipping	*i.v., weekly*	FDA conditional approval	
	Muscle	DMD	Viltolarsen	ASO, exon 53 skipping	*i.v., weekly*	FDA conditional approval	
	Muscle	DMD	Golodirsen	ASO, exon 53 skipping	*i.v., weekly*	FDA conditional approval	
	Muscle	DMD	NS-089/NCNP-02	ASO, exon 44 skipping	*i.v., weekly*	Phase III	NCT05996003
	Muscle	DMD	SCAAV9.U7.ACCA	U7-mediated exon 2 skipping	*i.v., single dose*	Phase I/II	NCT04240314
	Muscle	DMD	Dyne-251	ASO, exon 51 skipping	*i.v., monthly*	Phase I/II	NCT05524883
	Muscle	DMD	WVE-N531	ASO, exon 53 skipping	*i.v., every 2w*	Phase I/II	NCT04906460
	Muscle	DMD	AOC 1044	ASO, exon 44 skipping	*i.v.*	Phase I/II	NCT05670730
	Muscle	DMD	SQY51	ASO, exon 51 skipping	*i.v.*	Phase I/II	NCT05753462
RNA interference	Motor neuron	SOD1-ALS	Tofersen	ASO-mediated silencing	*IT, every 2-4w*	EMA/FDA approval	NCT04856982/NCT03070119
	Motor neuron	FUS-ALS	ION363 (Jacifusen)	ASO-mediated silencing	*IT, every 4-12w*	Phase III	NCT04768972
	Muscle	DMD	Ataluren	ASO-mediated silencing	*Oral, daily*	EMA, conditional approval	
	Muscle	FSHD	AOC 1020	ASO-mediated silencing	*i.v.*	Phase I/II	NCT05747924
	Muscle	DM1	DYNE-101	ASO- targeting DMPK	*i.v.*	Phase I/II	NCT05481879
	Muscle	DM1	AOC 1001-CS1	SiRNA- targeting DMPK	*i.v., every 1.5 m*	Phase II	NCT05479981
	Liver	ATTRv	Patisiran	ASO-mediated silencing	*i.v., every 3 m*	EMA/FDA approval	
	Liver	ATTRv	Vutrisiran	ASO-mediated silencing	*s.c., every 3 m*	EMA/FDA approval	
	Liver	ATTRv	Inotersen	ASO-mediated silencing	*s.c., weekly*	EMA/FDA approval	
	Liver	ATTRv	Eplontersen	ASO-mediated silencing	*s.c., monthly*	Phase III	NCT05071300
Cell therapy	Muscle	DMD	CAP-1002	Cardiosphere cells	*i.v., every 3 m*	Phase III	NCT05126758
	Muscle	DMD	DMD06-Mab	U7-mediated exon 51 skipping	*i.m*	Phase I	2023-000148-47
Other therapies	Nerve	CMT1A	PXT3003	Enzymatic substrates	*Oral*	Phase III	NCT04762758
	Nerve	CMT2 SORD	AT-007	Aldose reductase inhibitor	*Oral*	Phase II/III	NCT05397665
	Muscle	DMD	Vamorolone	Corticosteroid analogue	*Oral, daily*	FDA approval and positive opinion by EMA	
	Muscle	DMD	ATL1102	ASO targeting CD49d	*s.c., weekly*	Phase IIb	NCT05938023
	Muscle	DMD	RIMEPORIDE	NHE-1 inhibitor	*Oral, daily*	Phase II	
	Muscle	DMD	Givinostat	HDAC inhibitor	*Oral, daily*	Phase III	NCT02851797
	Muscle	DMD	TAS-205	HPGDS inhibitor	*Oral, daily*	Phase III	NCT04587908/jRCT2041200055
	Muscle	BMD	Vamorolone	Corticosteroid analogue	*Oral, daily*	Phase II	NCT05166109
	Muscle	BMD	EDG-5506	Muscle membrane stabilizer	*Oral, daily*	Phase II	NCT05291091/NCT06100887
	Muscle	FSHD	Losmapimod	MAPKs inhibitor	*Oral, daily*	Phase III	NCT05397470/NCT04004000
	Muscle	FSHD	RO7204239	Anti-Myostatin Ab	*s.c*	Phase II	NCT05548556
	Muscle	DM1	Tideglusib	GSK3 inhibitor	*Oral*	Phase II/III	NCT05004129
	Muscle	LGMD2I-R9	Ribitol	Enzymatic substrate	*Oral, daily*	Phase III	NCT05775848

*Ab* antibody, *ALS* amyotrophic lateral sclerosis, *AAV* adeno-associated virus, *ASO* antisense oligonucleotide, *BMD* Becker muscular dystrophy, *CMT* Charcot-Marie-Tooth, *DMD* Duchenne muscular dystrophy, *DM1* myotonic dystrophy type 1, *dys* dystrophin, *EMA* European Medicines Agency, *FDA* Food and Drug Administration, *FSHD* facioscapulohumeral muscular dystrophy, *GAN* giant axonal neuropathy, *HDAC* Histone deacetylase, *HPGDS* hematopoietic prostaglandin D synthase, *i.m.* intramuscular, *i.v.* intravenous, *IT* intrathecal, *MAPK* mitogen-activated protein kinases, *LGMD* limb girdle muscular dystrophy, *s.c.* subcutaneous, *SMA* spinal muscular atrophy, *SMARD* spinal muscular atrophy with respiratory distress syndrome

## Motor neuron disorders

### Spinal muscular atrophy (SMA)

5q SMA is an AR neuromuscular disorder caused by mutations in *SMN1* gene leading to defective expression of the survival motor neuron (SMN) protein. SMN is a ubiquitously expressed protein whose deficiency causes the abnormal development and subsequent degeneration of alpha motor neurons in the spinal cord [1]. The clinical picture is characterized by progressive weakness and atrophy affecting skeletal, respiratory, and bulbar muscles. The global prevalence is approximately 1:10.000 individuals, making SMA among the more frequent neuromuscular disorders. SMA encompasses a spectrum of severity, with its most severe form presenting in infancy and leading to significant disability and early mortality. Historically, SMA has been divided in different sub-types based on the age of onset and achievement of motor milestones [2]. SMA type 0 manifest antenatally, SMA type 1 between 0 and 6 months (patients never acquire the ability to sit), SMA type 2 between 7 and 18 months (patients never acquire the ability to walk), SMA type 3 between 1.5 and 10 years (patients usually acquire the ability to walk which is later lost), and SMA type 4 in adulthood. The disease severity largely (but not exclusively) depends on the number of copies of a paralogue gene called *SMN2*. *SMN2* differs from *SMN1* due to a single nucleotide variant that causes an abnormal splicing. This variant specifically produces the exclusion of exon 7, leading to the production of a truncated protein that is quickly degraded. Consequently, a single copy of the SMN2 gene typically generates only approximately 10% of the full-length SMN protein. Hence, a higher percentage of functional protein is produced in presence of a higher number of *SMN2* copies, contributing to the partial amelioration of the clinical phenotype. Other exonic/intronic *SMN2* variants that further affecting splicing may explain the mismatch observed in some patients between the number of *SMN2* copies and clinical severity [3].

Until few years ago the treatment of SMA was uniquely supportive, with limited beneficial effect achieved by the administration of β-adrenergic agonists in milder forms. However, the advances in the understanding of the underlying molecular mechanisms led to the development of targeted therapies, transforming the treatment landscape for this condition with 3 different compounds approved by US and European regulators (FDA and EMA) at the time of this review.

#### Gene therapies

Onasemnogene Abeparvovec (Zolgensma) was the first FDA and EMA-approved gene replacement therapy in the field of neuromuscular disorders. This involves a one-time intravenous infusion of a recombinant AAV9 virus containing a functional copy of the *SMN1* gene and is indicated for patients affected by SMA type 1 (EMA). Zolgensma was first tested in an open-label, phase I clinical trial in a small cohort of early-onset patients and has shown remarkable efficacy in prolonging survival and maintaining motor, bulbar and respiratory function [4]. Safety and efficacy findings were confirmed in larger cohort of patients and in real-world data [5]. Given the rapid deterioration of motor neuron, [1] it is not surprising that, in line with what observed with other compounds [6], the magnitude of the effect is greater when patients are treated in a pre-symptomatic stage [7, 8]. The main side effects of gene therapy are related to the immune reaction against the capsid. This is greater the higher the dose aministered. Elevated serum levels of liver enzymes may be observed after infusion but can be controlled by steroid conditioning, as well transient platelet reduction. Thrombotic microangiopathy has been anedoctally reported in real-world observations. Weight-based dosing still poses safety considerations due to higher viral load in heavier patients. Thus, studies are ongoing to prove safety and efficacy in children up to 21 kg (NCT04851873) and intrathecal administration of the product is been explored as a way to deliver higher doses of the vector directly to the central nervous system (CNS) [9].

#### Molecular therapies

Both Nusinersen (intrathecal) and Risdiplam (oral) counteract the exclusion of exon 7 of *SMN2* with different biological mechanisms [2]. Nusinersen (Spinraza; an ASO) achieved a significant milestone as the first drug to successfully complete randomized sham-controlled clinical trials in early onset SMA cases, leading to its FDA approval in 2016 [10]. Subsequent real-world data and insights from early access programs have confirmed Nusinersen’s safety and efficacy across a more extensive patient population [11, 12]. Furthermore, ongoing research is exploring the potential benefits of higher-dose Nusinersen administration (NCT04089566). Risdiplam (Evrysdi, a small molecule) was approved in the USA for administration across the entire spectrum of SMA patients, and the indication is about to be extended prior to 2 months of age also in other countries. The initial trial (FIREFISH) demonstrated a significant increase in patient survival and function in symptomatic SMA type 1 patients [13, 14]. Subsequently, efficacy and safety were proven also in patients with SMA type 2 and non-ambulant SMA type 3 (SUNFISH) aged between 2 and 25 years [15]. Importantly, Risdiplam exhibited a favourable safety profile and was well-tolerated in both trials. An open-label study designed to evaluate Risdiplam efficacy in pre-symptomatic patients under 6 weeks of age is ongoing (RAINBOWFISH) [16].

#### Other therapies

The use of β2adrenergic agonist (e.g., salbutamol/albuterol) may be considered in older patients affected by SMA type II and III, in view for its potential to increase *SMN2* protein levels and beneficial effect on the neuromuscular junction [17, 18].

#### Therapeutic considerations

The development of new therapies has radically changed the prognosis of patients affected by SMA. However, new challenges are emerging. It is likely that new phenotypes will appear following the increased survival of patients belonging to the most severe end of the spectrum. Preclinical studies showed that the lack of SMN protein exerts detrimental effects not only on motor neurons but also on other organs. The multisystem involvement in SMA in humans is still a matter of debate, [19] but this could theoretically indicate that a systemic drug should be preferable compared to a product which is administered intrathecally [20]. Gene therapy offers the advantage of a one-shot administration and systemic delivery, but its applicability at present is limited by dose-related side effects. While the use of combinatory strategies in under investigation (e.g., NCT05067790; NCT04488133), the choice of treatment is currently tailored on individual bases and there is no clear evidence indicating a superiority of a therapy over another. Only a systematic collection of real-world data considering possible confounding factors (e.g., genetics, definition of pre-symptomatic state etc.) will help improve the prescription of such treatments and clarify whether some aspects (e.g., bulbar function) are differently addressed by these products. There is strong evidence of a greater effect achieved in patients treated before the onset of symptoms, hence advocating the implementation of newborn screening for SMA.

### Amyotrophic lateral sclerosis (ALS)

ALS is an unrelenting disease marked by the degeneration of upper and lower motor neurons, leading to progressive muscle paralysis and, ultimately, fatal respiratory failure within 3–5 years [21]. Despite ALS being perceived as a monolithic entity, it represents a broad disease spectrum encompassing various clinical phenotypes likely caused by distinct pathogenetic mechanisms, all converging on the common outcome of motor neuron degeneration. Furthermore, the coexistence of frontotemporal dementia (FTD) within this spectrum adds an additional layer of complexity underscoring the intricate relationship between motor neuron degeneration and cognitive and behavioral changes. Over the past two decades, advancements in ALS genetics have witnessed exponential growth, providing robust insights into the underlying pathogenesis of this condition charting the course for more precise and targeted treatment approaches.

The identification of the Cu/Zn superoxide dismutase type-1 (*SOD1*) gene as the first causative factor of ALS in 1993 constituted a groundbreaking discovery in the genetics of this disease [22]. Mutations in *SOD1* account for 20% of familial ALS (*f*ALS) cases and 1–2% of sporadic cases (*s*ALS) [23]. The pathogenesis of the disease entails a gain-of-neurotoxic function [24]. Mutations in the *SOD1* gene cause an unstable, misfolded protein, resulting in intraneuronal accumulation and cellular damage. Additionally, this misfolded protein could spread via a prion-like trans-neuronal mechanism, leading to spatial dissemination inducing a relentless motoneuronal death [25].

Transactive response DNA-binding protein 43 (TDP-43), encoded by the TAR DNA-binding protein 43 (*TARDBP)* gene, is a multifunctional DNA/RNA binding protein. TDP-43 is recognized for its role in overseeing RNA processes, encompassing RNA splicing, mRNA transport, translation, and the regulation of non-coding RNAs [26]. In 2006, TDP-43 was identified as the main constituent of pathological cytoplasmic aggregates in ALS [27]. Following this discovery, *TARDBP* gene was identified as causal factors in ALS, contributing to 4% of fALS cases and less than 1% of sALS cases [3]. The cytoplasmic accumulation of TDP-43 is associated with a dual mechanism: the loss of TDP-43 function in the nucleus and the acquisition of toxic TDP-43 function in the cytoplasm, or a combination of both. Evidence from *TARDBP* knockout and TDP-43 overexpression models, have demonstrated that both TDP-43 deficiency and excessive TDP-43 can serve as causative factors in ALS [28–30]. Hence, modulating TDP-43 expression and/or subcellular localization holds promise as a potential therapeutic strategy for *TARDBP*-ALS patients.

In 2009, pathogenic variants of the Fused in Sarcoma (*FUS*) gene, were identified as causative factors in ALS [31]. FUS mutations contribute to approximately 4% of *f*ALS and a less than 1% of *s*ALS [23]. These mutations exhibit an AD inheritance pattern and lead to a juvenile aggressive form of the disease. *FUS* is a widely expressed RNA-binding protein primarily located in the nucleus, where it plays roles in DNA repair and RNA metabolism [32]. While the exact pathogenic mechanism of these mutations remains incompletely understood, there is substantial evidence supporting a toxic gain-of-function mechanism in *FUS-*ALS [33].

In 2011, the hexanucleotide GGGGCC repeat expansion (HRE) within the first intron of the *C9ORF72* gene was discovered to be causative for ALS [34, 35]. The *C9ORF72* HRE is implicated in roughly 40% of *f*ALS and 7% of *s*ALS, although substantial variability exists among different populations [36]. The pathogenesis of *C9ORF72*-ALS, although not fully elucidated, is underpinned by a combination of gain and loss of function mechanisms, as supported by substantial evidence. The HRE is instrumental in giving rise to three distinct pathological hallmarks within *C9ORF72*-ALS [37]. Firstly, it disrupts transcription, leading to *C9ORF72* haploinsufficiency, thereby compromising autophagic processes, deregulating autoimmunity, heightening cellular stress, and perturbing nucleocytoplasmic transport [38, 39]. Secondly, bidirectional transcription of the *C9ORF72* HRE generates transcripts that accumulate within cellular nuclei, sequestering RNA-binding proteins and giving rise to RNA foci [40, 41]. Thirdly, both sense and antisense transcripts of the HRE can serve as templates for the repeat-associated non-AUG (RAN) translation, resulting in the production of toxic poly-dipeptides. These dipeptides, prone to aggregation, have been identified in the CNS of individuals with *C9ORF72*- ALS and showed toxicity in cells and animal models [42–46].

#### Gene therapies

CRISPR/Cas9 targeted the *SOD1* gene in neonatal SOD1G93A mice using a modified AAV9 delivery system. Staphylococcus aureus-derived Cas9 and a single-guide RNA were administered through the facial vein, resulting in reduced SOD1 expression in the spinal cords. This intervention increased motor neurons, delayed disease onset, and extended survival in transgenic mice [47]. Subsequently, two studies reported similar reductions in SOD1 expression within the spinal cord and improved survival outcomes [48, 49]. In a study exploring the safety profile and exploratory efficacy outcomes, it was shown that an AAV carrying a microRNA (miRNA) targeting SOD1 can suppress the expression of the mutated *SOD1* gene in patients harboring *SOD1* mutations [50]. A single intrathecal infusion of AAV encoding SOD1-targeting miRNA was given to two patients. One patient showed a temporary improvement in right leg strength and reduced SOD1 levels in CSF, while the other maintained stable scores on a composite measure of ALS function for 12 months. The effectiveness of CRISPR/Cas9 in targeting (G4C2) repeat DNA or RNA has been recently proved [51]. These studies aimed to diminish the repetition of RNA transcription or mitigate the levels of RNA foci/DPRs, respectively. It is worth noting that a primary limitation of these investigations lies in the fact that the treatments were administered to mice at a young age before the onset of ALS symptoms. Consequently, it remains unclear how effective this treatment would be in older mice with manifested ALS symptoms. The use of gene editing techniques in ALS caused by the TARDBP and FUS genes has primarily been focused on enhancing the understanding of the pathogenetic mechanisms in cellular models, with limited therapeutic applicability data [52–54]. As is well-established, cognitive dysfunctions are observed in up to 50% of ALS patients, meeting diagnostic criteria for FTD in approximately 10% of cases (10.1136/jnnp-2013-307223). Consequently, when identifying therapeutic molecular targets and devising delivery strategies, it is essential to recognize that ALS is not solely confined to motor neurons. Neglecting this broader involvement may restrict therapeutic efficacy to the motor aspects of the disease, potentially lacking an impact on cognitive impairments.

#### Molecular therapies

Tofersen, an ASO, emerged as the foremost targeted therapeutic approach for *SOD1*-ALS [55, 56]. Administered intrathecally, Tofersen targets *SOD1* mRNA, degrading it and reducing SOD1 protein synthesis. It demonstrated an extended survival in a *SOD1*-mutation rodent model compared to the placebo-treated group [57]. Among *SOD1*-ALS patients, Tofersen reduced CSF SOD1 levels and achieved a significant decrease in plasma NfL levels. However, Tofersen failed to achieve statistical significance in reducing the decline in ALS Functional Rating Scale—Revised score (ALSFRS-R), which was the primary clinical endpoint of the 28-week phase III trial (NCT02623699). In the 52-week open-label extension study, patients who received early treatment with Tofersen exhibited significantly less deterioration in ALSFRS-R, respiratory function, grip strength, and patient-reported measures of quality of life compared to the placebo group. The limitations that may have led to an underestimation of the results could include the small sample size and the initiation of therapy at an advanced stage of the disease. In fact, studies are currently underway on presymptomatic and early-stage patients. An alternative strategy for addressing the pathogenic gain-of-function associated with *SOD1* in ALS involves the application of RNA interference (RNAi). This approach stands apart from ASOs due to the structural distinction of RNA molecules, characterized by their double-stranded nature. While double-stranded RNAs may exhibit enhanced resilience during delivery, their functional activity necessitates sequential enzymatic processing stages, contrasting with ASOs that are single-stranded and readily capable of direct target binding [8]. In the course of the RNAi process, RNA molecules execute the degradation of mRNAs within the cytoplasm via engagement with the RNA-induced silencing complex (RISC), ultimately resulting in the suppression of gene expression [58]. Several studies have proved the effectiveness of RNAi strategy in *SOD1* animal models, such as delaying disease onset and increasing survival [59–61].

ION363 (Jacifusen), an ASO, targets *FUS* intron 6 non-allele-specifically. In *FUS* mouse models, ION363 halted disease progression compared to placebo. A 25-year-old *FUS*-ALS patient with the *FUS* p.P525L mutation received compassionate-use ION363 over 10 months, starting 6 months after clinical onset when non-ambulatory and ventilatory support was needed. She had monthly intrathecal infusions with good tolerance and no serious adverse events. Pre-treatment, she lost ~ 5 ALSFRS-R score points monthly, which significantly slowed during therapy. Unfortunately, she died a year after treatment due to worsening ventilatory and bulbar dysfunction. In 2021, a Phase III clinical trial (NCT04768972) started to assess whether ION363 offers any clinical benefit in mitigating disease progression in symptomatic FUS-ALS patients. These individuals undergo intrathecal injections every 4–12 weeks over a 61-week period, followed by injections every 12 weeks during the open-label extension treatment phase, with the study anticipated to conclude in 2024 [33].

As an indication of the complex pathogenesis of *C9ORF72*-ALS, several ASOs targeting specific gene transcripts, such as BIIB078 and WVE-004, have proven ineffective in phase II clinical trials. An additional study provided proof of concept in a single human subject, demonstrating the efficacy and safety of intrathecal administration of Afinersen (ASO5-2) in suppressing *C9orf72* transcripts. This intervention resulted in an impressive 80% reduction in poly-dipeptide levels, while the individual maintained functional stability over an 18-month observation period [62]. Other approaches, such as RNAi, small compounds targeting (G4C2) expansions, antibody immunization against DPRs, interference with cell-to-cell transmission, clearance strategies for toxic DPRs, and inhibition of DPR production, have shown promise in animal and cellular models. Yet, their clinical applicability remains unproven at this stage [63–66]

#### Cell therapies

Stem cell therapy, derived from various sources, shows promise in preserving motor units through mechanisms like neurotrophic support and modulation of excitotoxicity and neuroinflammation. Preclinical findings support this, and ongoing phase I and II clinical trials in ALS patients indicate positive results in safety and tolerability. However, substantial improvements for ALS patients necessitate continued collaboration between basic and clinical researchers [67, 68].

#### Other therapies

Several studies have highlighted a substantial activation of immunity cells such as macrophages, microglia, and T cells in ALS. However, it is plausible that this activation is a secondary epiphenomenon resulting from massive neurodegeneration of the motor system rather than a fundamental mechanism of the disease’s pathogenesis [69]. Several pharmacological approaches, encompassing complement inhibition, eosinophil and sphingosine-1-phosphate receptor modulation, interleukin-2 receptor targeting, and diverse immunosuppressive regimens, as well as regulatory T-cell interventions, have been investigated in ALS trials, yet none have exhibited efficacy [70]. An exception is Masitinib, a tyrosine kinase inhibitor. Its mechanism involves targeting crucial pathways associated with neuroinflammation and immune response modulation. Encouraging results from a phase IIb/III clinical trial (NCT02588677) and subsequent investigations indicate a potential slowdown in disease progression among ALS patients as compared to placebo. Ongoing confirmatory study (NCT03127267) seeks to further substantiate these findings [71, 72].

Other therapeutic approaches are aiming to target free radicals and alleviate oxidative damage in motor neurons. Nicotinamide Riboside and Pterostilbene exhibit potential in addressing oxidative stress-induced damage, leading to increased survival and amelioration of ALS associated neuromotor function loss in SOD1G93A transgenic mice. Notably, further investigation is underway, with ongoing recruitment NO-ALS trial (NCT05095571) to delve deeper into their efficacy and mechanisms of action. Furthermore, the MICABO-ALS trial (NCT04244630) is currently investigating EH301, a combination of antioxidants (phase II). This trial aims to replicate previous positive findings showing substantial clinical improvement [73]. Intravenous Edaravone has been explored for its potential therapeutic efficacy in ALS. While the initial phase II trial showed positive results, these findings were not confirmed by the subsequent phase III study [74]. Nevertheless, a sub-analysis of efficacy targeting a specific subgroup of ALS patients demonstrated encouraging outcomes, which were considered adequate by regulatory authorities for the commercialization in ALS patients [74]. The approval of Edaravone prompted subsequent trial that verified the safety of the oral formulation, although efficacy data (NCT04569084) are pending [75]. Independent post-marketing studies have revealed divergent findings regarding the effectiveness of Edaravone, leading clinicians to express skepticism about the efficacy of this molecule [76–78].

The CENTAUR trial (NCT03127514) investigated Sodium Phenylbutyrate and Taurursodiol (AMX0035) for ALS. Taurursodiol enhances mitochondrial energy production, while Sodium Phenylbutyrate alleviates endoplasmic reticulum stress by upregulating chaperone proteins. Although the phase II trial showed a modest reduction in ALSFRS-R, positive outcomes prompted the ongoing phase III trial, PHOENIX (NCT05021536), with results anticipated soon [79]. Preclinical evidence suggests that RIPK1 plays a role in the progressive dysmyelination and axonal degeneration observed in ALS through the engagement of necroptotic machinery [80]. The ongoing phase II HIMALAYA trial aims to elucidate the efficacy and safety of the SAR443820 molecule in inhibiting this pathway and slowing down ALS progression.

## Inherited peripheral neuropathies (IPNs)

IPNs refer to a broad and heterogenous group of disorders characterized by a complex phenotype where the neuropathy is often one out of many different clinical manifestations [81, 82]. When the neuropathy is the predominant feature and both motor and sensory components are affected, it is usually referred as to Charcot-Marie-Tooth (CMT) disease, which also includes predominant motor forms (distal hereditary motor neuropathy, HMN, or distal spinal muscular atrophy, dSMA) [83] and predominant sensory neuropathies (hereditary sensory autonomic neuropathy, HSAN). Although very heterogenous, three main phenotypes are distinguished in CMT according to clinical and neurophysiological criteria: CMT1 or demyelinating forms, characterized by reduced motor conduction velocity at median nerves (MNCV) below 38 m/s as cut-off; CMT2 or axonal neuropathies with almost normal MNCV values higher than 38 m/s, but reduced compound motor action potential (CMAP). CMT neuropathies are now classified using an integration of clinical, neurophysiological, and genetic criteria. Of note, at least 120 responsible genes have been identified thus far, which encode a huge variety of proteins including structural proteins of the myelin, of the axonal cytoskeleton, transcription factors, regulators of intracellular trafficking, of mitochondrial function, of protein synthesis or homeostasis, and ion channels [84]. In the last three decades, in addition to advances in the clinical and genetic definition of the different forms, preclinical research has made significant progresses with the generation and characterization of animal and cellular models established from human iPSCs, which have been instrumental to study pathogenetic mechanisms and validate the effectiveness of therapeutic strategies [85–87]. Thus, even if no therapies are available to date for CMT patients, in the last few years numerous therapeutic strategies have been tested at preclinical level, some of which translated at clinical stage in phase 1–3 [85]. In this paragraph, we will provide an overview of these strategies and we will finally focus on the CMT1A neuropathy, due to the *PMP22* gene duplication, the most frequent mutation in CMT that accounts for 50–60% of all CMTs.

### Gene therapies

Gene replacement strategies have been tested at preclinical level for both demyelinating and axonal CMTs, where the genes of interest have been expressed using AAV9 [85, 88]. Following systemic delivery or via intracerebroventricular (i.c.v.) and intrathecal injections, the AAV9 serotype is mainly targeting neurons and axons. Schwann cells are reached at high efficiency if AAV9 particles are delivered via intranerve injection and with good efficiency using intrathecal injection.

The X-linked CMT1X neuropathy represents the second most common CMT form and is the consequence of mutations in the *GJB1* gene, which encodes the CX32 (connexin 32) protein [86]. CX32 is expressed by Schwann cells in the nerve as in other cells, where CX32 forms gap junction channels that allow fast communications of small molecules such as metabolites, ions and others across multiple layers of the Schwann cell plasma membrane. Both loss- and gain-of-function mutations have been reported for *GJB1*, but the majority are loss-of-function mutations, which are nicely modeled by the *Cx32* KO mouse. Proof-of-principle of AAV9-mediated gene replacement therapy has been obtained by intrathecal injection of AAV9 expressing CX32 under the Schwann cell specific promoter [89, 90].

AR CMT4J is due to the loss of the FIG4 phosphatase [91]. Delivery of FIG4 using i.c.v. injection at P1 (postnatal day 1) in the *Plt* (pale tremor) mouse model with complete loss of FIG4 resulted in a robust amelioration of the central and peripheral neuronal phenotype, which in this model is mainly neuronal [92].

Giant axonal neuropathy (GAN) is a very rare form of AR neuropathy caused by loss-of-function mutations in *GAN1*, which encodes an E3 ubiquitin ligase [87, 93]. GAN is characterized by enlarged axons which are abnormally packed with microtubules and intermediate filaments. Preclinical studies demonstrated that AAV-mediated GAN1 delivery reverted the enlarged axon phenotype and a scAAV9/JeT-GAN phase I/II clinical trial is ongoing for GAN with first patients already treated intrathecally (NCT02362438) [94].

Neurotrophin 3 (NT3) is one of the autocrine factors that Schwann cells secrete to survive and proliferate during development [95]. Sahenk et al., hypothesized that supplementation of exogenous NT3 could maintain terminal Schwann cells in a growth promoting state, thus overcoming the loss of regeneration capacity following the chronic progression of the neuropathy [96]. Intramuscular delivery of scAAV1.tMCK.NT-3 in different models of demyelinating or axonal CMT provided proof-of-principle of this approach that has been then translated in a phase I/IIa trial (NCT03520751).

AD CMT2D neuropathy is caused by heterozygous mutations in *GARS*, which encodes the glycyl-tRNA synthase. In vitro and in vivo data suggest a gain-of-function mechanism [87]. Morelli et al. provided evidence that RNAi specifically targeting *GARS* mutated alleles in different mouse models significantly ameliorated the axonal phenotype. RNAi was delivered in AAV9 particles either by i.c.v. or by intrathecal injection [97, 98]. Particularly for axonal/neuronal phenotypes, pre-onset early treatment was more efficient than post-onset later treatments as expected.

### Other therapies

Drug-based strategies have been tested at preclinical level for both demyelinating and axonal CMTs. Accumulation of misfolded proteins and activation of stress response in Schwann cells is thought to be at the basis of different forms of demyelinating CMT, such as the AD CMT1E due to point mutations in the *PMP22* gene and the AD CMT1B, associated with mutations in the *MPZ* (myelin protein zero) [86, 99–101]. MPZ contributes to myelin compaction by forming *cis* and *trans* interactions on different layers of Schwann cell plasma membrane [86]. Administration of curcumin to relevant mouse models for CMT1E and CMT1B has been found to alleviate cellular stress, decrease the UPR (unfolded protein response) and ultimately to reduce the severity of the neuropathy in these mice. A similar strategy is based on the administration of Sephin1/IFB-088, an inhibitor of GADD34, the phosphatase that activates translation by dephosphorylating the eIF2alpha translation initiator factor. In different CMT1B mouse models, IFB-088 treatment was able to improve the neuropathy by attenuating protein translation and alleviating cell stress [86, 102, 103].

The *SORD* (sorbitol dehydrogenase) gene is mutated in the AR CMT2 with predominant motor involvement [104]. When the SORD enzyme is lost, sorbitol accumulates in cells leading to toxicity. Aldose reductase is an enzyme that produces sorbitol from glucose and its activity can be inhibited by already FDA-approved compounds that are now under investigation in clinical trials along with natural history studies [105].

Heterozygous mutations in the *MFN2* gene (mitofusin 2) are associated with the axonal CMT2A neuropathy, which accounts for 20–30% of all axonal CMTs [106]. MFN2 is an outer mitochondrial membrane protein which mediates fusion between mitochondria. MFN2 seems to be also involved in mitochondrial transport. In vitro and in vivo studies suggested that mitochondrial abnormalities can be reversed by stabilizing the interaction between the normal copies of MFN2 and MFN1, homologous to MFN2 but expressed at very low levels in axons. Small molecules have been developed as agonist of mitofusin function showing promising results in mutant mice [107, 108].

Acetylation of tubulin is known to stabilize axonal microtubule network thus facilitating anterograde transport. Decreased tubulin acetylation has been shown in several models of axonal CMT2 neuropathies, thus suggesting that this mechanism can represent a commonality across different axonal forms independently on the specific pathogenetic mechanism [87]. Consistent with this, small molecules able to inhibit HDAC6 (histone deacetylase), an enzyme that deacetylases alpha tubulin, ameliorate the neuropathic phenotype in different CMT2 mouse models [109, 110]. Another promising approach as unifying treatment strategy for different axonal neuropathies consists in the drug-mediated inhibition of SARM1 activity [111]. When axons are severed, SARM1 is activated and rapidly hydrolases NAD + provoking a dramatic loss of ATP and mitochondrial dysfunction, leading to axonal degeneration. Mutant mice with loss of Sarm1 are protected from axon degeneration. Thus, inhibition of SARM1 activity has been thought to prevent or reduce axonal degeneration also in CMTs. Small molecule inhibitors are under development with one promising candidate, DSRM-3716 able to prevent axonal degeneration after axotomy in sensory neurons established from human iPSCs [112].

### The CMT1A neuropathy

CMT1A is a demyelinating generally slowly progressive neuropathy caused by 1.4 Mb duplication on chromosome 17p11.2 encompassing the *PMP22* gene, which encodes the peripheral myelin protein 22 (PMP22). As a consequence of an inequal crossing over event at the meiotic cell division, CMT1A patients carry three copies of the *PMP22* gene whereas the reciprocal event, the *PMP22* gene deletion, is associated with the HNPP neuropathy (Hereditary liability to pressure palsies) [86, 113]. A gene dosage effect has been proposed at the basis of the CMT1A Schwann cell pathogenesis. PMP22 proteins tends to misfold and aggregate and these events are physiologically resolved through the proteasome. In CMT1A patient cells, this mechanism is less efficient as proteasome is overwhelmed and blocked resulting in the activation of cell stress mechanisms. Animal models carrying several copies of *PMP22* recapitulate proteostatic stress with PMP22 accumulation and have also highlighted a role for PMP22 in Schwann cell differentiation during early stages of postnatal nerve development. A consistent number of therapeutic strategies tested at the preclinical level are aimed at reducing PMP22 levels in CMT1A using gene therapy and gene editing approaches as well as drugs, which target responsive elements in the *PMP22* promoter.

#### Gene therapy and molecular therapies to decrease *PMP22* expression

These strategies include ASO delivered subcutaneously; shRNA and miRNA expressed by AAV9 viral vectors delivered by intraneural injection and intrathecal injection, respectively; siRNA carried by squalene nanoparticles administered systemically and, finally, Crispr/Cas9 RNP (ribonucleoparticles) delivered by intraneural injection [85]. These approaches demonstrated efficacy in animal models, even if with some limitations due to the way of delivery and the translatability to CMT1A patients. How to control the degree of downregulation that could result in the HNPP phenotype remains to be assessed.

#### Other therapies to decrease *PMP22* expression

Drug mediated approaches able to reduce *PMP22* expression at preclinical level include onapristone treatment, an inhibitor of progesterone which stimulates *PMP22* gene expression and ascorbic acid, which inhibits adenylate cyclase and the production of cAMP, a potent *PMP22* expression inducer [114]. Due to toxicity, onapristone has not been tested in clinical trials. Of note, ascorbic acid, which was able to ameliorate the neuropathic phenotype in animal models, was not efficient in modifying the disease in several clinical trials where ascorbic acid was administered at different dosages, for different periods and in pediatric as well as adult cohorts of CMT1A patients [115–118].

#### Strategies to improve proteostasis

Other strategies are aimed at resolving proteostatic stress in Schwann cells [85]. These include Rapamycin, a known mTORC1 pathway inhibitor and autophagy activator with the aim of improving the clearance of aggregates in glial cells. Others are aimed at increasing the expression of chaperons that can improve the cytosolic trafficking of the PMP22 protein by using HSP90 inhibitors, a chaperon which stabilizes misfolded aggregated proteins. Finally, curcumin and Sephin1/IFB-088 have been tested in CMT1A cells as PMP22 aggregates can elicit UPR.

#### Other approaches

Inhibitors of HDAC6 have been tested at preclinical level also for CMT1A with the aim of delaying axonal degeneration that is the consequence of demyelination [119]. In CMT1A, the P2X7 channel is overactivated resulting in increased calcium level in Schwann cells leading to demyelination. Antagonist of P2X7 have been tested in cellular and animal models with improvement in myelination, but clinical trials were not envisaged due to toxicity of these compounds [120]. NRG1 (Neuregulin) type III on the axonal surface is one of the main signals regulating myelination in the PNS [121]. It has been hypothesized that overexpression of NRG1 type III can be beneficial for those CMT characterized by decreased myelination and demyelination, such as CMT1A and CMT1B, whereas this signal can be decreased in those characterized by excessive aberrant myelin such as autosomal recessive CMT4B and CMT4H [85]. Of particular relevance is the PTX3003 that is a combination of different compounds including baclofen, a GABA receptor agonist which reduces *PMP22* expression; naltrexone, believed to potentiate baclofen activity, and D-sorbitol, a metabolite involved in the polyol pathway thought to stabilize misfolded protein [122, 123]. This combination was selected using a system biology approach by screening repurposed drugs promoting differentiation and decreasing *PMP22* expression. A phase 2 study confirmed safety and tolerability of PXT3003 and showed improvement of CMT Neuropathy Score (CMTNS) and the Overall Neuropathy Limitations Scale (ONLS) using the highest dose for longer treatment period. A phase III clinical trial is ongoing to assess efficacy in CMT1A patients (NCT04762758).

### Hereditary transthyretin amyloidosis (hATTR)

Hereditary amyloidosis resulting from mutations in the TTR gene (hATTR) is a rare, progressively debilitating, and potentially life-threatening autosomal dominant multisystem disorder [124]. This condition results from the systemic accumulation of misfolded transthyretin, a transport protein for thyroxine and retinol. Clinical presentations vary, often showing initial symptoms related to peripheral nervous system involvement, such as small fiber neuropathy, autonomic dysfunction, and peripheral polyneuropathy. This diverse spectrum in hATTR underlines its complexity, not entirely explained by genetic diversity alone, despite some genotype–phenotype correlation [124, 125]. The most prevalent mutation worldwide, p.Val50Met, can manifest as either early-onset small fiber and autonomic involvement or late-onset “classic” peripheral polyneuropathy, primarily due to large fibers degeneration coexisting with cardiac amyloid deposition, typically remaining subclinical. Conversely, p.Val142Ile is the most frequent mutation in the US and is distinguished by its early and prominent cardiac manifestations. More than 120 pathogenic TTR mutations have been documented, each associated with a subtly distinct pattern of disease onset, symptoms, and progression.

TTR is a homotetrameric protein, with each monomeric unit being encoded by a small four exon gene. Each monomeric unit consists of 127 amino acid protein arranged in eight anti-parallel β-sheets. This characteristic structural arrangement partially accounts for the heightened amyloidogenic potential exhibited by this protein. Mutations within the TTR gene result in destabilization of the native tetrameric structure. Consequently, misfolded subunits tend to self-assemble into amyloid fibrils, which subsequently accumulate within various tissues, ultimately culminating in the development of disease. The deposition of amyloid derived from TTR in the peripheral nervous system appears to initiate at the level of sympathetic and dorsal root ganglia (DRGs) [126]. Over time, this deposition propagates along the course of the nerves [127]. However, the precise mechanisms underpinning neurodegeneration remain uncertain. Notably, direct mechanical influences contribute to the pathogenesis, particularly in early-onset patients and instances of localized nerve compression (e.g., the median nerve at the carpal tunnel or the ulnar nerve at the cubital tunnel). Conversely, in late-onset forms, neuroinflammatory and neurotoxic effects attributed to TTR aggregates play a pivotal role, as substantiated by in vitro studies [128].

Therapeutic strategies revolve around reducing the synthesis of mutant TTR, enhancing its stability to prevent misfolding and deposition, eliminating already deposited amyloid fibrils, or altering the defective gene entirely. A significant milestone in the management of hATTR was the advent of liver transplantation, which was first successfully performed in 1990. Subsequently, numerous patients have undergone this procedure, resulting in disease stabilization in many cases [129].

#### Gene therapies

NTLA-2001 is an in-vivo gene editing drug based on the clustered regularly interspaced short palindromic repeats and CRISPR-Cas9 system [130]. It is administered by intravenous infusion and comprises lipid nanoparticles containing mRNA for Cas9 protein and a single guide RNA targeting TTR. After hepatic uptake and translation, Cas9 binds to the guide RNA and forms a ribonucleoprotein that mediates a double strand DNA break in the TTR gene. Endogenous DNA repair mechanisms introduce insertions or deletions in the open reading frame, leading to frameshift mutations that prevent TTR synthesis. In a pivotal phase I study, a single administration showed dose-dependent reduction (up to 87% decrease) in serum TTR levels 28 days after infusion.

#### Molecular therapies

In 2018 Patisiran and Inotersen were approved by regulatory agencies for the treatment of hATTR. Patisiran is a double-stranded siRNA delivered intravenously via lipid nanoparticles, enhancing hepatic uptake. Inside hepatocytes, it targets both wild type and mutant TTR mRNA, inducing their degradation and inhibiting hepatic synthesis [131]. Inotersen, on the other hand, is a single-stranded ASOs administered subcutaneously. Following administration, it exhibits widespread tissue distribution, with notably high concentrations in the kidneys and liver. In hepatocytes, it interacts with nucleic TTR pre-mRNA, leading to degradation mediated by Ribonuclease H. Both drugs have demonstrated remarkable efficacy in reducing serum TTR levels and have proven effective in stabilizing disease progression [132, 133]. Similarly, Vutrisiran was the last siRNA-based drug to receive approval for the treatment of hATTR [134]. A phase III trial assessing the effectiveness of Eplontersen, a subcutaneously administered ligand-conjugated antisense drug designed to enhance hepatocyte uptake, has recently achieved its co-primary and secondary outcomes, and awaits results publication [135].

#### Other therapies

Doxycycline and tauroursodeoxycholic acid have been used off-label in hATTR based on preclinical evidence suggesting nonspecific anti-amyloid properties [136]. Removal of already deposited amyloid is an appealing approach, especially in the context of cardiomyopathy, and is being investigated with two monoclonal antibodies (NNC6019–0001 and NI006) that showed promising results in preclinical studies and phase I trials [137]. Diflunisal, a Non-Steroidal Anti-Inflammatory Drug, has found utility as an off-label treatment for TTR amyloidosis. Its application stems from its in-vitro capacity to stabilize TTR tetramers, thereby impeding the dissociation of misfolded subunits and the formation of amyloid aggregates [138]. A groundbreaking development was the approval of the first disease-specific drug, Tafamidis, by EMA in 2011, followed by its approval by the U.S. FDA in 2019. Tafamidis exerts its therapeutic effect by binding to one of the two thyroxine-binding sites of the TTR tetramer, thereby stabilizing the properly folded protein and preventing dissociation and amyloid fibril formation [139].

## Congenital myasthenic syndromes (CMS)

CMS are a group of neuromuscular disorders caused by pathogenic variants in genes encoding for proteins that are essential for the functioning of neuromuscular junction (NMJ) transmission*.* The proteins involved in CMS are usually clustered according to their localization, namely pre-synaptic, synaptic and post-synaptic; several different pathways are involved. These include (a) axonal transport (b) synthesis, recycling, storage and exocytosis of acetylcholine (ACh), (c) maintenance of the transmission between pre and post synaptic structures, (d) mutations within the muscle acetylcholine receptor (AChR), (e) maintenance of AchR clustering and stability of the synaptic cleft, (f) and protein glycosylation [140].

CMS usually present at birth or during early childhood, but adult-onset CMS mimicking acquired myasthenia gravis can be observed. The distinguishing features of CMS include exercise intolerance, fatigability, muscle weakness, and low muscle tone. Some patients may display myopathic features. Additionally, individuals may experience drooping eyelids (ptosis) with or without ophthalmoparesis, as well as respiratory and speech-related symptoms, joint stiffness (contractures), and abnormal spine curvature [141]. In rare instances, dysmorphic features may be present, and certain genes have been linked to CNS traits, like intellectual disability and seizures, or involvement of other organs [142]. At present, there is no curative treatment for CMS and no gene or molecular therapy have achieved human application. AAV-mediated gene replacement for *DOK7*-related CMS was beneficial in mice, but drug-development is still at preclinical stage [143].

### Other therapies

Treatment of CMS is currently aimed at alleviating symptoms. Acetylcholinesterase inhibitors are the most commonly used alone or in combination with 3,4-diaminopyridine (3,4-DAP), which increases Ach in the synaptic cleft acting at a pre-synaptic level via potassium channel blockage, or β-receptor agonists (ephedrine, salbutamol), which were shown to ameliorate the structure of the synaptic cleft [144]. In slow-channel CMS, namely those linked to specific mutations in AChR subunits, as well as in ColQ and DOK7 related CMS, pyridostigmine should be avoided and salbutamol could be considered. Fluoxetine and quinidine have been used in slow-channel CMS [145].

## Muscular dystrophies

### Dystrophinopathies

Dystrophinopathies are a spectrum of X-linked neuromuscular conditions caused by pathogenic variants in the *DMD* gene. This encodes for dystrophin, a protein with a structural role anchoring the actin cytoskeleton to the sarcolemma along with other proteins that together form the dystrophin-associated protein complex (DAPC). Duchenne muscular dystrophy (DMD) constitutes the most severe end of the spectrum, while Becker muscular dystrophy (BMD is a milder disease form with later onset and slower progression. Moreover, mutations in the *DMD* gene occasionally cause isolated X-linked cardiomyopathy [146] or may manifest in symptomatic female carriers [147]. DMD is the most common form of muscular dystrophy affecting around 20 per 100.000 live male births, [148] while the prevalence of BMD is fewer than 8 cases per 100.000 [149]. The primary effect caused by the lack of the dystrophin protein in skeletal and cardiac muscles is progressive muscle degeneration and necrosis, which is reflected in the changes observed on muscle histology. Disruption or disassembly of the DAPC results in a cascade of consequences that profoundly impact muscle cell functionality and are likely to act in parallel to generate muscle damage. These comprise susceptibility to muscle stress, NOS-dependent functional ischemia, increased reactive oxygen species production (ROS), calcium overload, mitochondrial dysfunction, inflammation, fibrosis, and inability to properly regenerate muscle [150].

Despite the early appearance of muscle weakness, affected boys can slowly acquire new motor skills up to the age of 7 years. Afterwards, the disease displays a progressive weakening of skeletal, respiratory, and cardiac muscles. In the absence of treatment, loss of ambulation (LoA) occurs by a median age of 10–12 years in “typical” DMD boys [151–154]. Thanks to the development of guidelines for care and management [155–157], the early initiation of corticosteroids (CS) treatment, and the optimal management of cardiopulmonary function, patients with DMD can now survive beyond their forties [158–161]. However, premature death may still occur during adolescence or early adulthood due to cardiac failure, fatal arrythmias, respiratory failure, and fractures-induced fat emboli. With the improvement of respiratory care, cardiac failure remains the leading cause of mortality in the older patients. At present there is no curative treatment for the disease. Dystrophinopathies are caused by a wide array of different type of mutations [162]. Deletions are the most common molecular defect (68–77%) [163, 164], followed by point mutations, duplications (11–13%) [163–166], and small rearrangements. While there is no clear correlation between the size of the deletion and the resultant clinical phenotype, the DMD vs BMD form can largely be predicted based on the *“reading frame rule”*: a severe, DMD phenotype is associated in ~ 90% of cases with out-of-frame pathogenic variants that induce a frameshift in the protein-coding sequence resulting in unstable RNA and subsequent nearly complete absence of dystrophin expression in muscles [163, 167]. By contrast, the presence of residual, smaller but functional dystrophin proteins resulting from in-frame deletions or duplications located in the middle of the gene and containing crucial domains can partially rescue the phenotype, hence being associated with BMD. The understanding of the genetic of Dystrophinopathies posed the basis for the development of several therapeutic strategies.

#### Gene therapies

Although the prospect of a non-mutation-specific therapy is intriguing, the large size of the *DMD* gene represents a major obstacle for currently used viral vectors, such as AAV (loading capacity < 4.7Kb) and lentiviruses (loading capacity up < 10kb). To overcome such limitations, different companies and labs developed micro or mini-dystrophins, which are truncated dystrophins lacking at least part of the central rod but designed to keep essential domains such as N-Term and Cysteine rich domain. A key point is that the large central rod domain is particularly resilient to large in-frame deletions, as demonstrated by mildly-affected patients harboring large in-frame deletions affecting up to 46% of the coding sequence [168]. The deeper understanding of the structure and role of dystrophin itself (e.g., nitric oxide and microtubule binding, protein stability etc.) helped implement the quality of these products, which are currently under investigation in Phase I/II trials. AAV serotypes 1, 6, 8, 9, rh10, and rh74 were found to have tropism for skeletal muscle and heart. FDA recently approved the first systemically delivered, AAVrh74-mediated delivery of a 138kDa micro-dystrophin in pediatric patients with 4 to 5 years of age. The main side effects of AAV gene therapy are linked to immunogenicity. Of note, these were not limited to the immune response generally expected in patients receiving AAV treatment (e.g., elevation of liver enzymes). Serious, T-cell mediated reactions observed in 5 patients treated with different micro-dystrophin constructs and vectors (*i.e.,* AAV9 and AAV rh74) suggest that the presence of mutations excluding exons coding for Hinge 1, (considered essential for the correct localization of dystrophin to the sarcolemma) and the first spectrin-like repeat domains, which are present in all the constructs but missing in these patients, likely provoked immune reactions against these nonself epitopes [169]. Following these observations, inclusion criteria for trials were amended. Additional concerns related to gene therapy are related to a) the ability of dystrophin constructs that are smaller than those observed in mild BMD cases and lack specific subdomains to fully fulfil their biomolecular role, b) the ability to transfect cardiac myocytes, c) the dilution in time of the product due to the limited efficacy of AAV vectors to target the stem cells reservoir [170].

#### Molecular therapies

Exon-skipping is a therapeutic strategy that employs ASO to induce alternative splicing, thereby bypassing mutated exons. This process aims to restore the reading frame of the dystrophin gene, effectively converting a DMD mutation into a BMD mutation. It has been estimated that exon skipping approaches might be applicable to 55% of DMD-causing mutations and 80% of DMD-causing deletions [171]. Various exon-skipping products have been developed, including 2′-O-methyl-modified RNA, phosphorodiamidate morpholino oligomers (PMO), and tricycloDNA antisense molecules. PMOs are uncharged compounds that pose challenges for in vitro cell introduction but exhibit high levels of systemic delivery and efficient exon skipping within dystrophic muscle tissue. To enhance cellular uptake, PMOs have been initially improved through conjugation with arginine-rich cell-penetrating peptides, resulting in peptide phosphorodiamidate morpholino oligomers (PPMOs). The safety profile of PMOs and PPMOs has generally been favourable. However, despite the appeal and applicability of exon-skipping techniques and the conditional approval of four ASOs, concerns persist regarding their delivery efficiency. Consequently, certain companies have developed PPMOs conjugated with fatty acid binders or specific antibodies (or antibody fragments, Fabs), to further enhance muscle penetration. Furthermore, there is ongoing exploration of multi-exon skipping to treat a broader patient population and ensure that the resultant transcript contains in-phase spectrin repeats. At present, the FDA has conditionally approved exon-skipping products targeting exons 51 (Eteplirsen), 53 (Vitolarsen and Golodirsen), and 45 (Casimersen) [172, 173]. These approvals have been granted based on observed changes in dystrophin expression in muscle tissue, while the full correlation with clinical benefits is still undergoing evaluation. Of note, a combined strategy using an AAV9 vector expressing U7 small nuclear RNAs has been explored to deliver targeted exon skipping in patient with exon-2 duplication [174, 175].

Around 10%–15% of patients with DMD have a nonsense mutation that induces a premature termination codon (PTC) in the mRNA, causing the ribosome to terminate translation and failing to synthesize the remainder of the protein. Ataluren (Translarna TM) was developed as a potentially safer alternative compared to other drug to induce stop codon read-through [176]. Due to the limitations of its efficacy data, EMA recommended the retraction of its approval, while real world data from registries may suggest a delay in LoA.

#### Cell-based therapies

Cell-based therapeutic approaches were initially explored to introduce full-length dystrophin into muscle tissues. However, significant challenges, primarily related to safety concerns and the limited distribution of cells following systemic administration, have yet to be fully addressed. Multiple cell types with multilineage or pluripotent potential have been investigated, including satellite cells (myoblasts), hematopoietic-derived cells, pericytes, and mesenchymal stem cells [177, 178]. Given the complexities associated with achieving satisfactory integration into affected muscles, current research in cell-based therapies is focusing on harnessing secondary effects rather than primarily rescuing muscle integrity. For instance, engineered mesangioblasts hold promise to facilitate exon skipping without the necessity for repeated infusions [179].

Cardiosphere-derived cells are believed to exert immunomodulatory, antifibrotic, and regenerative effects in individuals with dystrophinopathy and heart failure through the secretion of exosomes containing bioactive cargo. The HOPE-2 trial (NCT03406780) is a multicentre, double-blind, placebo-controlled Phase II clinical trial involving the repeated intravenous administration of CDCs. In a small subset of non-ambulatory patients, preliminary results suggest the ability to attenuate the progression of upper limb and cardiac impairment [180].

#### Other therapies

The cornerstone of DMD treatment is represented by the use of corticosteroids (CS). Although the first trials date back almost 50 years [181], only in the last two decades solid scientific evidence was gathered to build strong recommendation on their use and provide information of strengths and limitations of different dose regimens [182]. The precise mechanisms of CS effect remain unclear, but postulated actions encompass membrane stabilization, increase in total muscle mass and strength, stimulation of insulin-like growth factors, enhanced myoblast proliferation, reduced fibrosis, and attenuated inflammatory responses. Different regimes (daily vs intermittent) can be selected to balance efficacy with numerous side effects, but everyday administration yields higher results in terms of preservation of muscle function [183]. Treatment is usually started around 4–5 years of age and should be continued even after the loss of ambulation, due to potential beneficial effect on the preservation of upper limbs strength, cardiorespiratory function, and survival [152, 184–186]. There is strong evidence that CS can delay LoA by 2–4 years [151, 187]. A longer preservation of ambulation also results in later onset of both respiratory insufficiency and severe scoliosis [188]. Despite the beneficial for slowing disease progression, CS treatment is associated with a range of side effects that require careful monitoring and management. Vamolorone is a molecule that has been developed to reduce the burden of CS treatment. While retaining anti-inflammatory properties, this was designed to have fewer side effects compared to traditional CS used in DMD. The molecule showed promising results indicating an improved safety profile in terms of bone and growth-related morbidities and was recently recommended for marketing authorization by the EMA [189].

Considering the instability of the muscle membrane in DMD, an innovative therapeutic approach may involve the utilization of synthetic block copolymers as membrane stabilizing agents. This strategy aims to mitigate muscle damage by directly enhancing the stability of the dystrophin-deficient muscle membrane. Promising preclinical findings are paving the way for clinical trials involving the administration of P188 (NCT03558958).

Lastly, the role of Givinostat, a histone deacetylase inhibitor with potential anti-inflammatory activity, is being explored in Phase III trial (NCT02851797) and is being considered for the treatment of BMD.

Among products that failed to demonstrate a clinical benefit there are anti-oxidant (Idebenone—Sideros trial), anti-inflammatory molecule targeting the NF-κB pathway (Edasalonexent—PolarisDMD Trial), [190] anti-fibrotic monoclonal antibodies (Pamrevlumab—Fibrogen), and the repurposed drug tamoxifen (TAMDMD—NCT03354039). Myostatin inhibitors were thought to be a good candidate given potential to increase muscle bulk and counteract fibrosis. However, all the trials in human failed, possibly due to pre-existing downregulation of myostatin pathways observed in patients affected by neuromuscular disorders [191]. Upregulation of utrophin, a structural and functional paralog of dystrophin encoded by the *UTRN* gene that is activated in the absence of dystrophin, has been explored in preclinical models. So far, no products have been translated to humans and concerns remain regarding its potential effectiveness, given the lack of important domains.

### Facioscapulohumeral muscular dystrophy (FSHD)

FSHD is one of the most common inherited muscular diseases, with an estimated incidence of 5–12 affected individuals per 100,000 in the population [192]. Named for its distinctive muscle involvement pattern, FSHD commonly manifests with facial, scapular girdle, and proximal upper limb muscle impairment, marked by noticeable asymmetry. While most patients experience a gradual progression of muscle weakness over time, approximately 20% of patients exhibit a severe, disabling phenotype necessitating wheelchair use [193, 194]. The inheritance pattern is AD; however, the disease displays incomplete penetrance, and phenotypic variability and severity can be substantial both among family members and within individual patients. This phenotypic and penetrance heterogeneity is linked to genetic and epigenetic factors [195–197].

The pivotal chromosomal locus in the pathogenesis of FSHD is the D4Z4 macrosatellite tandem repeat array situated in the subtelomeric region of the long arm of chromosome 4 (4q35). In the somatic cells of healthy individuals, this region is transcriptionally repressed [198, 199]. Under normal conditions, this region consists of 10 to 100 tandem repeat units, each measuring 3.3 kb [198]. Each of these repeats contains a retrogene housing the full open reading frame of double homeobox 4 (*DUX4*) [198, 200, 201]. The distal sequence of the D4Z4 region exhibits two sequence variants, denoted as 4qA and 4qB, with the key distinction being the exclusive presence of a polyadenylation signal (PAS) in the 4qA variant [202]. The pathogenetic mechanism underlying FSHD1, the most common form of the disease, involves a de-repression of the *DUX4* gene [194]. This occurs due to a deletion/contraction of the repeat units within D4Z4, typically ranging from 1 to 9 repeats, in the presence of the permissive 4qA allele [198, 199]. The contraction induces a partial loss of methylation within the D4Z4 region, resulting in chromatin relaxation and facilitating the transcription of *DUX4*. The presence of the PAS contributes to maintaining the stability of *DUX4* mRNA and, consequently, its expression in muscle cells. The triggering of *DUX4* expression in experimental settings, either through overexpression or inducible methods, has been shown to induce cytotoxic effects in diverse somatic cell types. However, the principal pathway governing DUX4-induced cytotoxicity remains uncertain [203, 204]. FSHD2 accounts for approximately 5% of patients with the phenotype and is characterized by the presence of an allele within the normal range of D4Z4 repeat units. However, the de-repression of *DUX4* is facilitated by the presence of mutations in certain chromatin repressor genes, including *SMCHD1* (in 85% of cases), *DNMT3B*, and *LRIF1* [205–207]. In this intricate context, many factors come into play, including D4Z4 repeat units, DNA methylation, mutations in *SMCHD1*, chromatin remodeling proteins and epigenetic factors, which influence the FSHD phenotype and progression of the disease.

Despite significant advancements in understanding the underlying mechanisms of the disease, there are currently no specific therapies for FSHD. To mitigate disease progression, rehabilitative physical activities are recommended, along with the use of orthoses aimed at improving patient functionality [194].

#### Gene therapy

Silencing of DUX4 through a CRISPR technology can be achieved essentially by two approaches: 1) editing (CRISPRe), which utilizes a functional Cas9 to alter the genomic sequence, and 2) inhibition (CRISPRi), which uses an enzymatically inactive “dead” Cas9 (dCas9) fused to a transcriptional or chromatin repressor. CRISPRi would have advantages on CRISPRe in FSHD, as it does not damage the genome and has limited off target effects being a repressor of regions that we would like to keep silent.

Since 2016, a pioneering study showcased the viability of utilizing the CRISPR/dCas9 technology as transcriptional inhibitor to specifically target the Krüppel-associated box (KRAB) zinc finger proteins and other transcriptional repressors at the D4Z4 locus. This approach effectively reduced the transcription of DUX4 in myocytes, offering early evidence of the potential of CRISPR/dCas9 in modulating the expression of key genes implicated in FSHD [208]. Subsequently, further gene editing approaches have been applied in vitro, focusing on inhibiting the transcription of DUX4 exon 3 PAS, although with unsatisfactory results [209]. The failure of this approach is due to the identification of an additional PAS, located upstream, responsible for the residual DUX4 transcript. Consequently, the subsequent successful steps have been directed towards a multifunctional approach, combining direct targeting of the DUX4 PAS and utilizing the dCas9-KRAB inhibitor to restore the heterochromatin state at the D4Z4 locus [209]. In an independent investigation, the adenine base editor system was employed alongside Cas9-nickase to instigate an AT to CG conversion within the DUX4 PAS in immortalized myoblasts derived from FSHD1 and FSHD2 patients. This method successfully induced mutations in the PAS, thereby preventing the expression of DUX4 [210]. In a recent study, a novel strategy was employed to target loci within repeats, which posed technical challenges due to the presence of multiple CRISPR–Cas9 binding sites and the potential for generating off-target effects through multiple DNA breaks [211]. The authors generated and optimized miniaturized cassette to be effectively packed in AAV vectors and to drive a muscle-specific expression of a smaller dCas9 orthologue targeting different epigenetic regulators of DUX4 expression. This innovative approach demonstrated efficacy in reinstating the epigenetic (permanent) repression of DUX4 with minimal impact on neighboring D4Z4 repeats or predicted off-target genes [211]. Furthermore, the method successfully achieved DUX4 repression and targeted gene modulation in a FSHD transgenic mouse model, albeit with a modest effect. In conclusion, while gene therapy holds promise for addressing the molecular intricacies of FSHD and has shown encouraging preclinical results, extensive research is essential to overcome delivery challenges, improve targeting specificity, and optimize treatment efficacy before its widespread clinical application.

#### Molecular therapies

Several recent studies have demonstrated the potential therapeutic application of ASO in the treatment of FSHD, by reducing the transcription of DUX4 and consequently its expression in muscle tissue. Compared to mice treated with a placebo, those treated with ASO exhibited improvements in muscle histology, reduced fibrosis and inflammation, as well as enhanced motor performance [212–216]. Similarly, the intramuscular delivery of miR-405 using a AAV vector in mice co-transduced with AAV-DUX4 has proven effective. This method successfully diminished DUX4 protein concentration and mitigated DUX4-induced muscle pathology by directing the DUX4 mRNA transcript towards degradation [217, 218]. Oligonucleotides designed to specifically target the antisense sequence at the 5ʹ end of U7 small nuclear RNAs, integral components of the small nuclear ribonucleoprotein complex crucial for the 3ʹ end processing of histone pre-mRNAs, were engineered to selectively interfere with the maturation of DUX4 pre-mRNA. Application of this modified agent resulted in a substantial reduction in DUX4 transcript levels within FSHD muscle cells. An additional advantage of this approach, in comparison to conventional ASOs and RNAi-based therapies, lies in the sustained activity of the redirected small nuclear ribonucleoprotein complex targeting DUX4 [219]. The FORTITUDE trial (NCT05747924), a Phase I/II study, was designed to explore both the safety and the efficacy of AOC 1020. AOC 1020 is an innovative antibody-oligonucleotide conjugate, incorporating a DUX4-targeting siRNA linked to a humanized antibody directed against transferrin receptor 1, optimizing its delivery to muscle tissues. The trial is actively recruiting patients with FSHD1 or FSHD2.

A last alternative approach is to block DUX4 recruitment of histone acetyltransferases p300 and CREB binding proteins to its target foci to avoid gene expression and DUX4-downstream cascade. This can be reached with p300 specific inhibitors (small molecules) or decoy DUX4 binding sites, which sequester endogenous DUX4 and prevent it from binding to and activating its target genes.

#### Other therapies

Albuterol, a β2 Adrenergic agonist, has shown beneficial effects on muscle mass and volume in placebo-controlled studies conducted in the early 2000s, despite not demonstrating efficacy on the primary outcome [220–222]. It was subsequently shown that the effect of β2 Adrenergic agonists might not be confined to their trophic effect on muscle but could involve a repressive mechanism on DUX4. Additional molecules that have demonstrated in preclinical studies a repressive effect on the *DUX4* gene include the inhibitors of the Mitogen-Activated Protein Kinase (MAPK) family, particularly the p38 and p38β isoforms [223, 224]. Eighty patients were therefore enrolled in the placebo-controlled REDUX4 phase 2b trial (NCT04003974). Losmapimod, a p38 inhibitor, was administered orally for 48 weeks. Although the molecule fell short of achieving the primary outcome, which aimed to demonstrate a reduction in DUX4 expression in muscle biopsies, it did reveal notable differences compared to the placebo-treated group across various secondary clinical and muscle MRI endpoints. No safety concerns were identified during the clinical trial [225]. The REACH study (NCT05397470), a placebo-controlled phase 3 trial, is currently ongoing. The enrollment of the planned 260 patients has been completed, but the results are not yet available. Finally, some evidence suggests additional targetable pathways to reduce DUX4 transcription, such as the inhibition of bromodomain-containing protein 4 (BRD4), or the regulation of Matrin 3 (*MATR3*) [226]. However, studies in FSHD animal models are not yet available. MANOEUVRE (NCT05548556) is a placebo-controlled Phase II trial investigating GYM329, an anti-myostatin antibody specifically designed to target skeletal muscles with the potential to enhance their size and growth. The trial is actively enrolling [227].

### Limb girdle muscular dystrophies (LGMD)

Recognized in 1954 as a separate entity, LGMD identifies patients showing onset of muscular dystrophy within the third decade of life, with weakness and atrophy predominantly in the proximal muscles at four limbs, sparing of facial muscles, and progressive course. The disease can be inherited as AD or AR, and the number of genes involved has been progressively raised until the number of 39, while a recent ENMC workshop revised this classification and the nomenclature [228]. To be considered a form of LGMD the condition must be described in at least two unrelated families with affected individuals achieving independent walking, elevated serum creatine kinase (CK), showing degenerative changes on muscle imaging, and have dystrophic changes on muscle histology. Albeit a similar clinical phenotype, LGMDs do not share a common pathological mechanism that would distinguish them from other forms of muscular dystrophy. Moreover, while some genes encode for proteins involved in the transmembrane connection of the muscle cytoskeleton to extracellular matrix, other have completely different molecular mechanism thus impeding at present a common unifying therapeutic strategy. The most frequent forms are related to mutations in gene *CAPN*, *DYSF, ANO5* and *FKRP*, while in pediatric patients are mainly involved genes causing sarcoglycanopathies, dystroglycanopathies, calpainopathies.

Except for physical therapy and prevention of cardiomyopathy, there is no treatment at present for any LGMD. Prednisone has been sporadically used mainly in sarcoglycanopathies, which are characterized by consistent inflammatory infiltrates in muscles [229]. Accordingly, recent preclinical findings showed that intermittent prednisone dosing reduced muscle damage and fibro-inflammatory infiltration in murine models of dysferlin and γ-sarcoglycan LGMD [230]. A subsequent exploratory study on 19 LGMD patients (including *CAPN3*, *DYSF*, SGCG, *SGCB*, *SGCD*, *FKRP*, *TTN*, and *ANO5*) receiving once-weekly prednisone at 0.75–1 mg/Kg for 24 weeks resulted safe and showed reduction of CK levels and a trend in improvement of motor performances [231].

#### Gene therapies

Preclinical studies have shown significant results in the use of recombinant AAV-mediated delivery of small size LGDM genes in rodents. Most results were observed for sarcoglycan genes. The rAAVrh74 vector containing a codon-optimized human *SGCG* transgene showed significant protein expression in skeletal muscle and heart of Sgcg-null mice, along with improvement of muscle histology, muscle force and motor functions [232]. Similarly, Sgca-null mice treated with the full-length human *SGCA* driven by the same vector (and a muscle-specific promoter) showed robust expression of the protein in the sarcolemma, improved the histopathology of limb and diaphragm muscles, and ameliorated motor functions and CK levels [233]. A similar strategy with the single stranded AAVrh74.tMCK.hCAPN3 transgene was effective in reverting the phenotype of the Capn3-null mice, [234] without the cardiotoxicity observed in previous treatment protocols [235]. Also *FKRP* mouse models were ameliorated by AAV9 delivery of mouse/human *FKRP*, including glycosylation of α-dystroglycan in heart and skeletal muscle, muscle histology and motor functions [236, 237]. However, a study posed the attention of possible muscle toxicity of FKRP overexpression [238]. Recently developed new optimized construct for FKRP with untranslated region (UTR) modifications and miniaturized muscle CK enhancer/promoter was delivered with AAV6, AAV9 and AAVMYO1 vectors showing good efficiency on the dystrophic phenotype (AAV6) and no toxicity (AAV9/AAVMYO1) on muscle phenotype [239].

Larger gene as *DYSF* has been instead delivered by dual-AAV system with overlapping cDNA sequences [240]. Results in mice showed some mitigation of the disease although data did not reach significance and the number of transduced myofibers was estimated at 35%.

In human, isolated limb infusion gene transfer has been performed with the scAAVrh74.tMCK.hSGCA construct in *SCGA* patients through the femoral artery with the dosage of 1 or 3 × 10^12^ vg/kg/limb. Ambulant patients showed modest α-sarcoglycan protein expression in muscle and conflicting functional outcomes [241]. Clinical studies with systemic delivery of *FKRP*, *SGCA* and *SGCB*, *DYSF* are ongoing.

#### Molecular therapies

Exon skipping through ASOs has been considered a useful mechanism also to treat LGMD. Proof of principle studies have been performed in ad-hoc generated mouse models such as *SGCG*, [242] or using patient-derived human cell lines, including patient carrying mutation causing *SGCG* [243]or *DYSF* LGMD [244]. Exon skipping strategy has been also proven for other LGMD mouse models, such as *DYSF*, [245] and *SGCG* [246].

#### Cell-based therapies

Cell therapy studies for LGMD are limited to experimental models. While transplantation of healthy myoblasts showed significant number of dysferlin positive myofibers in Dysf-null mice, [247] these cells did not migrate far from their injection site, thus several injections per muscle would be necessary, making this approach not feasible. Again, the most significative result is related to the use of mesoangioblast stem cells in the Sgca-null mice. Parental administration of congenic healthy stem cells resulted in significant amelioration of skeletal muscle histology, motor function and re-expression of the missing α-sarcoglycan protein [248].

#### Other strategies

A number of molecules have been tested in preclinical models of LGMD, some of them showing amelioration of the phenotype. Dysf-deficient mice displayed reduction of muscle fat infiltration by Ezetimibe treatment, a cholesterol reducing drug [249]. Similarly, recombinant human galectin-1improved muscle repair and function and reduced the inflammatory milieu when administered to Dysf-deficient mice [250]. Interesting results were obtained by six months administration of ribitol in mouse mutants for *FKRP* [251]. Ribitol is an important constituent for the functional maturation of α-dystroglycan and matriglycan impaired in *FKRP* disease. Results showed significant rescue in the functions of skeletal, respiratory, and cardiac muscles in a dose-dependent manner. Ribitol administration has been also used to ameliorate gene-therapy results in preclinical studies [252]. Molecules tested in clinical trials in humans did not show significant results so far, including the myostatin inhibitor domagrozumab recently reported in phase Ib/IIa, open-label study in FKRP patients [253].

### Congenital muscular dystrophies (CMDs)

CMDs comprise a heterogeneous group of genetic disorders primarily affecting the skeletal muscle and often associated with multisystem features [254]. The clinical picture is characterized by hypotonia and muscle weakness leading to a delay or arrest in the attainment of gross motor milestones; symptoms occur before the achievement of independent ambulation. Such definition provides a pragmatic boundary with LGMD, with many genes that are allelic for both conditions. The severity of muscle involvement is variable, but muscle biopsy usually displays dystrophic features and CK are more commonly elevated [255]. A classification of CMDs based on the localization of the protein defects defines a) CMDs related to defective structural proteins of the basal membrane or extracellular matrix (ECM), b) CMDs related to primary defects in dystroglycan (DG) or α-dystroglycan glycosylation, c) CMD related to defects in proteins of the edoplasmic reticulum (ER), d) CMDs related to defects in proteins of the nuclear envelope, and e) a wider group including newly recognized forms associated with proteins implicated in ER—Golgi trafficking, disorders affecting mitochondrial and lysosomal morphology and function, and muscular dystrophies with multisystem involvement and neurodegeneration [256]. With more than 35 genes identified so far, the overall prevalence of CMDs is 0.6–0.9 per 100.000 [257–259]. The most common forms are dystroglycanopathies (DGP; 12–25%), Collagen 6 related disorders (COL6-RD) (12–19%), laminin-alpha 2 related dystrophies (LAMA2-RD; 10–37%), and selenoprotein N related myopathy (SEPN1-RM; 11.65%). Due to a founder mutation, Fukuyama CMD (FCMD) is the most common form in Japan [260–262].

At present, treatment for CMDs is supportive and no drug is approved. In following section, we will offer a summary of the most promising therapeutic approaches getting closer to clinical application. A detailed review of all forms of CMDs, treatment strategies was recently published [256].

#### Gene therapies

The replacement of a defective gene is seen as the optimal strategy for many AR disorders. In CMDs however, this is not always feasible due to the large size of genes exceeding the loading capacity of currently used AAV vectors (i.e., *LAMA2* and *COL6A3*). Replacement is also not an option when disease mechanism derives from toxic gain of function mutations. At present, there are only two Phase I/II trials evaluating the efficacy of gene therapy in patients harbouring mutations in *FKRP (*causing both DGP and LGMDR9*)*, although these are exclusively recruiting individuals with a LGMD phenotype (NCT05224505 and NCT05230459). In general, dystroglycanopathies pose several challenges to treatment. For instance, while pre-clinical studies suggested that the overexpression of the glycotransferase encoded by *LARGE1* could enhance α-DG glycosylation also when the defect resided in other genes (*POMT1* and *POMGNT1),* [263], such change was not observed in presence of other genetic defects. Moreover, there are concerns regarding the potential toxic effect of α-DG hyper-glycosylation [264]. Finally, CNS plays a pivotal role in numerous dystroglycanopathies, and the prospect of therapeutic interventions administered post-development yielding a favorable impact on this aspect appears unlikely [265, 266].

Gene addition is seen as a possible strategy for LAMA2-RD. LAMA2-RD arises from pathogenic variants in the gene encoding the alpha-2 subunit of Laminin-211 (Lm-211), also known as merosin [267]. In patients with Lm-211 deficiency, upregulated laminin isoforms Lm-411 and Lm-511, sharing beta and gamma chains but with different alpha chains, are insufficient substitutes due to limitations in integrin-binding, poor alpha-DG binding affinity, and lack of the LN domain essential for polymerization. Strategies to overcome these constraints include Miniagrin, enhancing laminin binding to alpha-DG, and LN-domain nidogen-1 (αLNNd), enabling polymerization of alpha LN domain-lacking laminins. Co-expression of these molecules in animal models yielded promising results, restoring basement membrane stability and enhancing muscle function [268–271]. Of note, both products can be loaded to AAV vectors, hence raising hope for translation to humans.

The upregulation of laminin-alpha1-containing isoform Lm-111, which shares homology with Laminin-alpha2, has also shown potential in rescuing muscle and nerve phenotypes. However, challenges remain, as Lm-111 is only expressed during embryonic life, and gene size constraints limit its use in AAV vectors. Alternatives involve delivering exogenous human recombinant Lm-111 through infusions, with some concerns regarding dosing, biodistribution, and human toxicity. Lastly, another elegant approach is achieving CRISPR-dCas9-mediated upregulation of Lm-111, although feasibility in human remains a concern [272–274].

#### Molecular therapies

The modulation of gene expression through the utilization of ASO or RNAi represents a promising approach for addressing dominant negative and gain-of-function mutations. This approach holds relevance in those severe cases of COL6-RD caused by autosomal dominant mutations that usually locate close to the N-terminal region, including a recurrent deep intronic pathogenic variant in intron 11 of COL6A1 [275–278]. Since haploinsufficiency does not cause disease, a knock-down of the gene is considered safe.

In LMNA-RD, mutant lamins exert a dual negative impact: aberrant nuclear localization and diminished lamin levels. The precise mechanisms through which these abnormalities lead to muscle disease remain incompletely understood. Since most cases arise from toxic gain-of-function mutations, several research groups are actively developing therapeutic approaches to silence these toxic mutants. Such strategies involve siRNA or genome editing techniques, such as CRISPR/Cas9. In theory, a combined approach targeting the silencing or knock-down of abnormal transcripts while simultaneously replacing the defective gene could be employed. However, the optimal threshold of lamin A/C expression remains a subject of investigation, as both excessive and insufficient expression can detrimentally affect muscle cells.

Lastly, in the case of FCMD, a remarkable 80% of Japanese patients share a distinctive founder mutation that disrupts mRNA splicing, resulting in an aberrant protein with atypical amino acid composition at the C-terminus. This leads to abnormal protein localization from the Golgi apparatus to the endoplasmic reticulum (ER). Given the nature of this genetic defect, ASOs designed to target splicing modulating regions hold promise as a mutation-specific therapeutic strategy for FCMD [279].

#### Other strategies

Investigational products aimed at common pathogenic pathways hold the theoretical advantage of their applicability in more than one condition. Based on pre-clinical data suggesting a role of proapoptotic pathways in the generation of damage in LAMA2-RD and a beneficial effect observed in mice after the administration of the inhibitor of the Siah1-mediated nuclear translocation of GAPDH (named Omigapil), a 12-weeks, phase 1 clinical trial was completed in 2019 (NCT01805024) on patients affected by LAMA2-RD and COL6-RD [267]. While the compound was safe and well-tolerated, follow-up was too short to provide information regarding its efficacy. The development of the product in CMD was however discontinued.

A recently discovered important stage in the synthesis of the ligand-binding section of α-DG involves the addition of a tandem ribitol 5-phosphate (Rbo5P) structure to the sugar chain of α-DG [280]. This process is facilitated by ISPD and two Rbo5P transferases. The use of ribitol has demonstrated benefits in preclinical models and could potentially serve as a therapeutic avenue for patients carrying pathogenic variants in *ISPD*, *FKTN*, and *FKRP* [266]. A Phase III Placebo-controlled study aims to assess the effectiveness and safety of BBP-418 (Ribitol) in individuals diagnosed with LGMD2I (*FKRP)* is ongoing (NCT05775848).

Considering the presumed antioxidative and anti-ER stress properties associated with SEPN1, ER stress inhibitors have been explored as a potential treatment for SEPN1-RM. A preliminary clinical trial utilizing N-acetylcysteine was concluded in 2020 (NCT02505087), but the outcomes are not yet available.

## Myotonic dystrophies

This is a dominant inherited, multi-systemic, disease due to the expansion of the CTG triplet repeat in the *DMPK* gene for type 1 myotonic dystrophy (DM1) and the CCTG-tetranucleotide sequence in the *CNBP* gene for the DM2. DM1 is characterized by weakness in facial and distal limb muscles plus myotonia, while DM2 mainly affects proximal limb muscles and myotonia is often less evident. DM1, and in general with lower frequency and severity in DM2, also can present with non-muscular signs and symptoms including cognitive impairment, cataract, insulin resistance, infertility and endocrine dysfunction, cardiac arrhythmia, sleep disorders and hypersomnia, and dysphagia. While DM1 onset can be at any age, including neonatal and infancy (more severe forms), DM2 onset is only in adulthood [281, 282].

In both cases, the gene expansion has deleterious downstream effects in many different cells of the body, thus explaining the multi-organ disease. This mechanism is clear and well documented for DM1, while it is still not completely understood for DM2. In DM1, the production of an abnormal RNA (CUG repeats of abnormal *DMPK* transcripts) accumulates in the nucleus (RNA foci) and disrupts normal cellular function with a gain-of-function mechanism [283]. RNA foci dysregulate essential proteins, such as Muscleblind-like 1 and 2 (MBNL1/2) factors and CUGBP Elav-Like Family Member 1 (CUGBP1 or CELF1). The dysregulation of MBNL1/2 and CELF1 is responsible for the spliceopathy, that is the changes in the alternative splicing of hundreds of genes thus explaining the multisystemic characteristic of DM1 symptoms. Accordingly, animal models with long CTG repeat expansions replicate the disease [284]. Recently, miRNA dysregulation was suggested to contribute to MBNL1/2 protein depletion, as well as the dysregulation of other signaling pathways such as AKT, AMP-activated protein kinase (AMPK), and glycogen synthase kinase 3 beta (GSK3β) [285].

At present, the only treatments available for DM1 and DM2 are related to prevent myotonia with anti-epileptic drugs or the more effective mexiletine, an antiarrhythmic molecule blocking sodium channels [286]. This molecule is currently used in clinical practice as was shown to significantly reduce the frequency and severity of myotonia [287], but did not provide benefit in 6-min walk distance after 6 months of treatment [288]. Mexiletine has also shown the ability to downregulate *DMPK* mRNA levels, suggesting additional function in DM1 [289]. Although the use of Modafinil to treat hypersomnia in DM1 patients was not found to be effective [290], there is general consensus between experts suggesting that this drug can be useful in selected patients [291]. Other available treatments are related to the development of specific symptoms in the different target organs, and thus aimed at controlling cardiac, endocrine (e.g., hyperglycemia), and gastrointestinal manifestations.

Drugs directed against the pathogenesis of the disease are under development. A few different approaches have been developed to excise, block the expression, or modify the instability of CTG repeats with the intent to specifically target the mutant allele, its RNA product, or its downstream signaling pathways.

### Gene therapies

Gene editing is the mechanism of choice to target and delete the expanded (toxic) repeats. The advantage of this approach is that the DM1 mutation can be eliminated from the genomic DNA, preventing the toxic downstream effects. Several nucleases were tested to induce double-strand breaks in in vitro and small animal models showing variable results [292]. Better results were observed with the CRISPR/Cas9 technology to excise DM1 expansion in mouse models, [293] or patient-derived myoblasts, fibroblasts or IPS cells; reviewed in [294]. Tissue-specific and time restricted Cas9 activity will constitute a promising tool for reducing the risk of unintended genomic effects still limiting this technology.

### Molecular therapies

ASOs have been designed with two different aims: (1) to block MBNL1 interaction by binding to CUG repeats or (2) to induce the degradation of CUG-expansion-containing DMPK mRNA, as the stabilization of the repeat to postpone the onset or slow the progression of the disease. Different types of ASO have been tested in human cells or animal models with interesting results in the last years. For example, already in 2011 Nakamori et al. showed that CAG-repeat ASO that bind CUG-expanded RNA was able to suppress the expansion instability in DM1 models [295]. Many subsequent preclinical studies were performed and have been recently reviewed [296]. A similar strategy to target CUG expansion was achieved with RNA-i by siRNA or miRNA delivered by viral vectors [297, 298].

An alternative way to tackle MBNL1 sequestration is to use an engineered RNA binding domain of the protein itself as a decoy for CUG-expansion to reverse the toxicity of the mutant transcripts [299].

Clinical trials in human have been performed or are ongoing for both ASOs and RNA-interference strategy. Recently, a phase I/IIa trial with the ASO Baliforsen showed that the drug was generally well tolerated but with low efficacy [300], probably due to low muscle drug concentration, as generally observed for other ASOs in DMD. Even in this case, new strategies to increase ASO concentration in muscles, such as conjugation with specific antibodies, fatty acids or proteins, are under evaluation in clinical studies.

### Cellular therapies

Cell therapy studies in DM1 are a few. One potential limit might be the observation that donor-derived engrafted cells (nuclei) acquire toxic RNA foci containing MBLN1 sequestration and abnormal alternative splicing. This suggests that toxic CUG repeat-containing RNA may exit the endogenous nucleus and traffic to other nuclei in the syncytial myofiber, thus exerting pathological effects [301].

### Other strategies

Many molecules have been or are being tested for DM1 including new and repurposing drugs [302]. Among others, some have reached clinical evaluation and are briefly described below. Metformin, a biguanide antidiabetic drug, was shown to rescue multiple aspects of DM1. Metformin can correct DM1-related alternative splicing defects, alleviates muscle cell senescence and mitochondrial dysfunction, reduces the risk of developing cancer and improves mobility in DM1 treated patients [302]. A phase II clinical trial showed the efficacy of metformin on mobility in 40 patients with DM1 [298], while a larger study is on-going. Erithromycin, as the prototype of small molecules to inhibit the interaction between MBNL1 and CUG expansions has been successfully used in vitro and in mouse models of DM1 [303]. A clinical study in DM1 patients is ongoing. Preclinical studies with GSK3β inhibitors, such as lithium and Tideglusib gave positive results [304, 305]. Tideglusib was also tested in congenital and childhood-onset DM1 patients in a phase II study: the drug was safe and showed some improvement in neuromuscular and cognitive symptoms [306].

## Congenital myopathies (CM)

CM are a heterogeneous group of genetic disorders usually characterized by congenital hypotonia and weakness that typically exhibit stable or slowly progressive clinical course. Of note, the clinical spectrum ranges from milder forms (mainly with childhood but also adult onset) to severe neonatal cases, with involvement of skeletal, extraocular, respiratory, and bulbar muscles leading to severe disability and early death. Cardiac involvement is observed only in patients harbouring mutations in specific genes (e.g., *TTN, MYH7*). CMs have been historically defined by the histopathological findings described on muscle biopsy. The most common categories are (a) congenital myopathies with cores (central core or multi-minicore), (b) nemaline myopathies (defined by the presence of distinct rod-like inclusions called nemaline bodies), (c) and centronuclear myopathies. Historical classifications also included Congenital fibre type disproportion and Myosin storage myopathy [307]. Collectively, CM have a prevalence of 1:26.000 [308, 309] and up to 30 causative genes identified so far. Importantly, not only mutations in different genes can cause the same muscle pathologies, but mutations within the same gene can cause different clinical and histopathological findings. The pathophysiological mechanisms underlying CMs are mainly related to excitation–contraction coupling, intracellular calcium homeostasis, membrane trafficking and remodelling, and sarcomeric filament assembly and interaction [310]. To date, no approved treatment exists for CMs but there is hope for patients coming from both symptomatic and targeted strategies [311, 312].

### Gene therapies

X-linked myotubular myopathy (XLMTM) is a rare monogenic disease due to mutations in the *MTM1* gene that cause centronuclear myopathy. The clinical spectrum is wide including symptomatic female carriers, but 80% of male individuals manifest with a severe clinical phenotype. This presentation is characterized by significant prenatal and neonatal features, including polyhydramnios, reduced foetal motility, neonatal muscular weakness, hypotonia, and respiratory insufficiency. Motor developmental milestones are markedly delayed, and most affected individuals do not attain independent ambulation. Weakness often involving facial and extraocular muscles is severe. Respiratory compromise is nearly universal, with the majority of affected individuals necessitating continuous ventilatory support. Based on successful preclinical studies [313], a phase I/II open-label, ascending-dose study was performed to evaluate AT132, an AAV8-delivered gene therapy (NCT03199469). While preliminary data suggested potential clinical benefit on both motor and respiratory function [314], the study was halted for the death of 4 patients with ongoing severe cholestatic liver dysfunction [311]. Cholestasis was then recognized as an important feature of the disease and studies are ongoing to establish how to improve safety [315].

### Molecular therapies

Within the group of centronuclear myopathies, *MTM1, DNM2* and *BIN1* are interconnected due to the role they play in membrane trafficking. Preclinical studies showed that the downregulation of DNM2, whose levels are increased also in BIN1 and MTM1-related centronuclear myopathies, resulted in clinical and histological improvements [311, 316]. Based on these findings, a phase I/II, dose escalation trial testing the molecule DYN101 (an ASO directed against human DNM2 RNA), was performed on patients affected by XLMTM and DNM2 centronuclear myopathies. However, the trial was stopped due to safety issues. Alternative strategies (such as AAV-delivered shRNA or CRISPR/Cas) have been explored in mice [317, 318]. An further approach that prompted positive results in murine models of *MTM1* and *DNM2* related CM was the upregulation of amphiphysin 2 (encoded by *BIN1*), which is a negative regulator of *DNM2* [319].

### Other therapies

Another therapeutic strategy with potential benefit on multiple conditions is the use of a repurposed drug called Tamoxifen. Its beneficial effect on muscle function was demonstrated in preclinical models of BIN1, DNM2 and MTM1 related centronuclear myopathies [320, 321]. The TAM4MTM, a phase I/II double-blinded study to determine the safety and efficacy of tamoxifen therapy for XLMTM is currently ongoing (NCT04915846).

RYR1-related myopathies, allelic to the malignant hyperthermia susceptibility (MHS) trait, are caused by both autosomal dominant (milder) and recessive forms. Mutants in *RYR1*, encoding for the type 1 ryanodine receptor (an intracellular calcium release channel), cause abnormal sarcoplasmic reticulum calcium leak and increased activity of calcium-activated proteases. Hence, molecules aimed at stabilizing have been tested in preclinical models, [322] and in a Phase 1 clinical trial (NCT04141670). On the other hand, the use of the antioxidant N-acetylcysteine failed to meet primary endpoints in a trial conducted on patients harbouring *RYR1* mutants (NCT02362425). Results on patients affected by *SEPN1* related myopathies (which span from multi-minicore CM to rigid spine CMD) are still pending (NCT NCT02505087).

Lastly, there is evidence that in some patients with confirmed CM a transmission defects in the neuromuscular junction can be present [323]. Interestingly, whilst CM and CMS are distinct groups of disorders, there are in some cases overlapping clinical features, such as fatigable muscle weakness. Features suggestive of NMJ abnormalities include, alone or in combination, positive response to AChE inhibitors, abnormal repetitive nerve stimulation and increased jitter on single fibre electromyography [324–328]. Of note, long term anti-cholinesterase treatment has been shown to detrimentally affect end-plate structures and neuromuscular transmission; in CMS patients harbouring AChR mutants the initial beneficial treatment effects of AChE inhibitors can diminish over time. The addition of β2-adrenergic agonists to anti-cholinesterase treatment has been shown to rescue the loss of postsynaptic folding caused by long-term pyridostigmine treatment in AChR deficient mice [144]. Beneficial effects of a combinatory treatment, whether linked to the above mechanism or to the anabolic effects exerted by β2-adrenergic agonists, have been demonstrated in CMS patients with AChR deficiency [329]*,* and in anecdotical CM cases, even in the absence of clear neurophysiology abnormalities [326].

## Summary and conclusions

Although significant therapeutic results in neuromuscular disorders are still limited to SMA and hATTR neuropathies, a relevant number of molecules and products are under evaluation in preclinical and clinical studies for the whole spectrum of genetic neuromuscular disorders. The paradigm that genetic disorders are untreatable conditions has therefore dramatically changed, suggesting that in few years we will likely have numerous drugs available for various forms of inherited neuromuscular diseases. However, several issues persist. The efficient delivery of products to the precise cell compartment, including effective targeting of stem cells, still presents significant challenges. Moreover, it’s important to note that gene transfer might not always be feasible, particularly for extremely large genes, when utilizing AAV vectors due to the limited cargo capacity of this vector. Finally, advanced tools such as gene editing still maintain off-target toxic effects that limit their application.

## Data Availability

Not applicable.

## Abbreviations

AD: Autosomal dominant
ALS: Amyotrophic lateral sclerosis
AR: Autosomal recessive
ASO: Antisense oligonucleotide
BMD: Becker muscular dystrophy
CK: Creatine kinase
CMS: Congenital myasthenic syndromes
CMT: Charcot-Marie-Tooth
CSF: Cerebrospinal fluid
CNS: Central nervous system
DMD: Duchenne muscular dystrophy
ECM: Extracellular matrix
EMA: European Medicinal Agency
ER: Endoplasmic reticulum
FDA: US Food and Drug Administration
FSHD: Facioscapulohumeral muscular dystrophy
HMN: Hereditary motor neuropathy
HSAN: Hereditary sensory autonomic neuropathy
iPSC: Induced pluripotent stem cell
LGMD: Limb girdle muscular dystrophy
LoA: Loss of ambulation
NfL: Neurofilament light chain
NMJ: Neuromuscular junction
siRNA: Small interfering RNA
SMA: Spinal muscular atrophy
SMN: Survival motor neuron
UPR: Unfolded protein response
