# Correction: Molecular mechanisms and therapeutic strategies for neuromuscular diseases

**DOI:** 10.1007/s00018-024-05305-0

**Published:** 2024-07-26

**Authors:** Alberto Andrea Zambon, Yuri Matteo Falzone, Alessandra Bolino, Stefano Carlo Previtali

**Affiliations:** 1 Division of Neuroscience, Institute for Experimental Neurology, IRCCS San Raffaele Scientific Institute, Inspe, Milan, Italy; 2 Neurology Department, San Raffaele Scientific Institute, Milan, Italy; 3 Vita-Salute San Raffaele University, Milan, Italy

**Cellular and Molecular Life Sciences (2024) 81:198**

10.1007/s00018-024-05229-9

In the original version of this article, the given and family names of Alberto Andrea Zambon, Yuri Matteo Falzone, Alessandra Bolino & Stefano Carlo Previtali were incorrectly structured. The name was displayed correctly in all versions at the time of publication.

The original article has been corrected

